# A population of *in silico* models identifies the interplay between Nav 1.8 conductance and potassium currents as key in regulating human dorsal root ganglion neuron excitability

**DOI:** 10.12688/f1000research.74551.1

**Published:** 2022-01-27

**Authors:** Oliver J. Britton, Blanca Rodriguez

**Affiliations:** 1 Department of Computer Science, University of Oxford, Oxford, OX1 3QD, UK

**Keywords:** DRG neurons, hDRG neurons, pain, Nav 1.8, computational modelling, population of models

## Abstract

**Background:** The Nav 1.8 sodium channel has a key role in generating repetitive action potentials in nociceptive human dorsal root ganglion neurons. Nav 1.8 is differentiated from other voltage-gated sodium channels by its unusually slow inactivation kinetics and depolarised voltage-dependence of activation. These features are particularly pronounced in the human Nav 1.8 channel and allow the channel to remain active during repolarisation. Gain-of-function mutations in Nav 1.8 have been linked to neuropathic pain and selective blockers of Nav 1.8 have been developed as potential new analgesics. However, it is not well understood how modulating the Nav 1.8 conductance alters neuronal excitability and how this depends on the balance of other ion channels expressed by nociceptive neurons.

**Methods:** To investigate this, we developed a novel computational model of the human dorsal root ganglion neuron and used it to construct a population of models that mimicked inter-neuronal heterogeneity in ionic conductances and action potential morphology

**Results:** By simulating changes to the Nav 1.8 conductance in the population of models, we found that moderately increasing the Nav 1.8 conductance led to increased firing rate, as expected, but increasing Nav 1.8 conductance beyond an inflection point caused firing rate to decrease. We found that the delayed rectifier and M-type potassium conductances were also critical for determining neuronal excitability. In particular, altering the delayed rectifier potassium conductance shifted the position of the Nav 1.8 inflection point and therefore the relationship between Nav 1.8 conductance and firing rate.

**Conclusions:** Our results suggest that the effects of modulating Nav 1.8 in a nociceptive neuron can depend significantly on other conductances, particularly potassium conductances.

Research highlights **Scientific benefits**

• Software and model allow simulation of human dorsal root ganglion (hDRG) neuron electrophysiology incorporating human-specific data at action potential (AP) and ion channel levels where possible.
• Population of models incorporates observed experimental variability in hDRG neuron AP biomarkers and mimics variability in ion channel densities, allowing the effects of this inter-neuronal variability to be simulated.
• Use of computational modelling allows large numbers of experimental conditions to be simulated rapidly and for voltage traces and individual ionic currents in each simulation to be simultaneously recorded and analysed.

**3Rs benefits**

• These simulations have the potential to replace experiments using animal DRG neurons, particularly experiments for hypothesis generation and for studying electrophysiological mechanisms.
• These simulations can also be used alongside existing experimental approaches early in drug development to refine target identification and candidate selection, further reducing the need for animal studies.
• The current model can be iterated on and improved as new human-specific data becomes available, increasing the accuracy of the model and the range of animal studies it could potentially replace.

**Practical benefits**

• Many simulations can be run in a short amount of time, allowing rapid investigation of many different simulated experimental conditions.
• The models, population of models approach and simulation runner are implemented within a Python software package that is freely available and open source. Python itself is widely used by the scientific research community.

**Current applications**

• Studying hDRG neuron AP electrophysiology and the roles of different ionic currents in these neurons incorporating the effects of inter-neuronal variability in ion channel conductances.
• Simulating the effects of ion channel block and enhancement, and of ion channel mutations, in hDRG neurons.

**Potential applications**

• Studying propagation of action potentials in an extended model including the full hDRG neuron geometry.

## Introduction

The sodium channel Nav 1.8 is selectively expressed in small diameter dorsal root ganglion (DRG) neurons and has an important and unique role in pain signalling, due to its slow and depolarised kinetics of inactivation [ Akopian *et al*., 1996; Sangameswaran *et al*., 1996] and fast recovery from inactivation [ Dib-Hajj *et al*., 1997] compared with other voltage-gated sodium channels. Human Nav 1.8 has particularly slow inactivation and larger persistent current compared to rodent Nav 1.8 [ Han *et al*., 2015]. Gain-of-function mutations in Nav 1.8 contribute to painful peripheral neuropathy [ Faber *et al*., 2012; Huang *et al*., 2013; Han *et al*., 2014], and Nav 1.8 has been shown to support repetitive firing and the characteristic broad shoulder of DRG neuron action potentials [ Renganathan *et al*., 2001; Blair and Bean, 2002]. Nav 1.8 has also been identified as a potential therapeutic target, due to its effects on DRG neuron excitability, and selective blockers of the channel have been developed [ Jarvis *et al*., 2007; Payne *et al*., 2015]. However, to date, there has only been one clinical trial of a selective Nav 1.8 blocker, which was an unsuccessful trial of PF-04531083 [ Payne *et al*., 2015] for post-surgical dental pain.

It is therefore important to better understand how Nav 1.8 affects the excitability of human DRG (hDRG) neurons. However, the effect of an ion channel on neuronal electrophysiology depends not only on its individual kinetics and expression level but also on the background ensemble of other ion channels expressed in the neuron. For example, in DRG neurons expressing Nav 1.8, a gain-of-function Nav 1.7 mutation caused hyper-excitability while in sympathetic ganglion neurons without Nav 1.8 the same mutation causes hypo-excitability [Rush *et al*., 2006]. Understanding how variation in multiple ionic currents will interact with each other, not just on a qualitative level (whether a channel is expressed or not) but on a quantitative level (the level of expression of each channel), is challenging. This is an important consideration though, as ion channel densities have been shown to have high inter-neuronal variability [ Schulz *et al*., 2006]. They can cause different neurons to respond differently to the same experimental conditions, *e.g.* application of the same concentration of a drug. Pathological conditions also substantially alter expression of multiple channels in DRG neurons [ Black *et al*., 2004; Cao *et al*., 2010; Zhao *et al*., 2013].

In this study, we investigate the effects of modulating the Nav 1.8 conductance (GNav 1.8) on hDRG neuron excitability using computational modelling and simulation. We hypothesised that altering the Nav 1.8 conductance would be positively correlated with firing rate, but that the quantitative change in firing rate would depend on the balance of other conductances in a particular model. To test this hypothesis, we develop a novel model of the hDRG neuron based on human-specific ionic currents. We construct a population of 328 experimentally calibrated human DRG neuron models [ Britton *et al*., 2013; Marder and Taylor, 2011; Prinz *et al*., 2003/ 4] that integrate data from electrophysiological recordings of human DRG neurons at the cellular [ Davidson *et al*., 2014] and ion channel levels [ Han *et al*., 2015; Vasylyev *et al*., 2014; Huang *et al*., 2014]. Each model in the population shares the same underlying kinetics and equations for the eight ionic currents in the model, but has a different set of ionic conductances, reflecting inter-neuronal heterogeneity in ion channel densities. Importantly, every model in the population produces action potential behaviour in range with the experimental variability reported in human DRG neurons by Davidson *et al*. [2014] when simulated with equivalent protocols and measured with eight action potential parameters.

This study advances the replacement of animal DRG neurons for research into human DRG neuron electrophysiology by developing the first computational model of human DRG neuron electrophysiology and providing open-source software to run simulations using the model. This software allows interested users to begin using the model with the population of models approach described in this study for modelling inter-neuronal variability. The main situations where we think our model would be particularly suited to replacing the animal DRG neuron experiments are for hypothesis generation, as many different experimental conditions can be rapidly simulated and analysed; and for detailed analysis of ionic current behaviours as each current can be output and visualised.

## Methods

### Overview of modelling approach

In this study, we use a non-standard modelling approach based on experimentally calibrated populations of models [ Britton *et al*., 2013; Marder and Taylor, 2011]. This methodology, which originated in neuroscience [ Prinz *et al*., 2003, 2004; Marder and Taylor, 2011], has also been used extensively to model the electrophysiology of cardiac cells [ Britton *et al*., 2013; Passini *et al*., 2017], but to our knowledge this is the first time it has been used to study DRG neurons. This approach allows us to integrate the observed experimental variability in hDRG neuron action potential (AP) parameters [ Davidson *et al*. 2014] into the modelling process, instead of the standard approach where a single model parameterised to reflect a typical neuron is used [ Marder and Taylor, 2011].

Here we explain the population of models methodology. For more detail, see [ Britton *et al*., 2013; Marder and Taylor, 2011; Muszkiewicz *et al*., 2016]. The main idea is to create a group of neuronal models that all have different underlying conductance values (representing inter-neuronal variability in ion channel densities) that, when simulated, all generate voltage traces with AP biomarkers that are within the range of values observed experimentally in hDRG neurons.

To create our population of models, we first construct a baseline model. This model is a Hodgkin–Huxley-type neuronal AP model [ Hodgkin and Huxley, 1952] comprising a set of equations describing the changes in membrane voltage and ionic current gating variables over time. We then select parameters in the model to be varied to represent inter-neuronal variability. In this study we make the assumption that ion channel expression varies from neuron to neuron but that the structure of each ion channel type does not. Therefore, we randomly vary all conductances in the baseline model but leave the kinetics parameters of each ionic current constant. Through random variation of all conductances we obtain a pool of candidate models, each with a different set of conductances. We simulate each model to find its rheobase, record its voltage trace and from this trace calculate AP biomarkers. We then calibrate the population by comparing each AP biomarker in each model to the experimental range for that parameter. If any AP biomarker for a model is outside the experimental range, the model is rejected. If all tested AP biomarkers are within their experimental ranges then the model is accepted. The final population of models consists of only the accepted models. All models in the population have a different set of conductances but each model is in range with experimental data on AP parameters of *ex vivo* hDRG neurons.

### Baseline model

For this study, we chose to model the human dorsal root ganglion neuron soma, rather than the axon or full DRG neuron geometry. This is because the soma was the section of the neuron that was patched and recorded from in both the experimental data we used to calibrate the population [ Davidson *et al*., 2014] and in the vast majority of DRG neuron electrophysiological studies, due to its large size compared with the axon. Our aim was to create a model that could be directly compared against results from the experimental system from which data are available.

We developed a baseline model, which was a single model with no variability in ionic conductances. The baseline hDRG model contains eight ionic current models: INav 1.7 [ Vasylyev *et al*., 2014]; INav 1.8 [ Han *et al*., 2015]; INav 1.9 [ Huang *et al*., 2014]; IKdr [ Sheets *et al*., 2007]; an A-type potassium current (IKA) [ Sheets *et al*., 2007]; an M-type potassium current (IKM) [ Maingret *et al*., 2008]; a leak potassium current (IKleak); and a hyperpolarization-activated current (Ih) [ Kouranova *et al*., 2008]. All current models used a Hodgkin–Huxley formulation.

The kinetics (voltage dependencies of activation and inactivation, and time constants) of each ionic current were not varied in this study, while all eight conductances were simultaneously varied. These choices are based on the assumptions that: 1) kinetic parameters depend primarily on ion channel structure, which as a simplifying assumption for modelling we assume will not vary between different DRG neurons; 2) conductances represent the density of each ion channel type in the cell membrane, and these vary substantially between neurons [ Schulz *et al*., 2006].

The soma was modelled as a single cylindrical compartment with length=30 μm, diameter=46 μm, and cytoplasmic resistivity=1 Ω/cm following the values used in the DRG modelling study by Choi and Waxman [Choi and Waxman, 2011]. A cylindrical geometry was used in line with how the NEURON modelling software represents all neuronal compartments. The specific membrane capacitance was set to the commonly used value of 1 μF/cm ^2^. The internal and external ionic concentrations were fixed to match the experimental conditions used by Davidson *et al.* [2014] with extracellular [Na ^+^]=145 mM, intracellular [Na ^+^]=5 mM, extracellular [K ^+^]=3 mM, and intracellular [K ^+^]=135 mM.

The equations for the baseline model can be found in the supplementary material and source code is provided in the data supplement.

### Experimental data and stimulus parameters

We used data on experimental AP biomarkers of *ex vivo* human DRG neurons from Davidson *et al*. [2014] to calibrate the population of models against experimental data. This dataset included data from recordings of 141 *ex vivo* human DRG neurons from five organ donors.

Each neuron’s soma was recorded under two stimulus protocols, an 800-ms step stimulus and a 500-ms ramp stimulus. In both cases, stimulus current was increased in steps of 50 to 100 pA until AP threshold was reached for that neuron, and AP biomarkers were calculated from traces at that stimulus current amplitude.

**Measuring AP properties**

We used eight AP biomarkers to quantify the electrophysiological properties of hDRG neuron APs: AP threshold voltage; AP peak voltage; AP slope maximum; AP slope minimum; AP full width; after-hyperpolarisation time constant; resting membrane potential; and rheobase. Each biomarker was calculated as follows:

**Rheobase**

Rheobase was calculated for each model by running step current simulations with increasing stimulus current amplitudes in increments of 100 pA from 0 pA to up to 5000 pA until the first simulation in which one or more action potentials were detected. The stimulus amplitude of that simulation is the rheobase for that model.

**AP threshold voltage**

Following the approach used by Davidson *et al*. [2014], AP threshold voltage was calculated from a ramp current simulation and defined as the voltage during the upstroke of an action potential at which the voltage-time gradient first surpasses 5 mV/ms.

**AP peak voltage**

AP peak voltage was calculated as the peak voltage attained during an action potential.

**AP slope maximum and minimum**

AP slope minimum and maximum was calculated as the maximum voltage–time gradient and minimum voltage–time gradient during an action potential.

**AP full width**

AP full width for an action potential was calculated as the duration between the time at which the voltage first crossed the AP threshold voltage from below, and the time at which the voltage first crossed the AP threshold voltage from above.

**After-hyperpolarisation time constant**

The time constant (τ) of the after-hyperpolarisation of an action potential was calculated by fitting the after-hyperpolarisation to the following single-exponential model with a curve fitting algorithm and extracting the value of τ:

$$A\;e^{-t/\tau }+c$$

The after-hyperpolarisation of an action potential was defined as the period from the minimum voltage that occurred after the AP peak, up to the point at which the voltage gradient exceeded 5 mV/ms or 50 ms had elapsed, whichever came first.

**Resting membrane potential**

The resting membrane potential of a model was defined as the minimum voltage that occurred during the last 10% of a simulation in which no stimulus current was applied.

### Sampling and calibrating the population of models

To create the initial pool of candidate models, all conductances in the baseline model were simultaneously and randomly sampled in a range of 0–2-times the baseline model value for that conductance (Extended data - Equations_and_conductance_densities.docx – Conductance densities table) to create 20,000 candidate models with the same ionic currents and equations but different conductance values. Conductance sampling was performed using Latin hypercube sampling ( McKay *et al*. 1979), which is a technique that evenly samples each conductance without exhaustively sampling all possible conductance combinations. We used Latin hypercube sampling as sampling all possible combinations of eight conductances and simulating the resulting models is not computationally feasible, even if each conductance is sampled coarsely. The range of 0–2-times baseline was chosen to allow a broad range of different conductance profiles within the population. Previous studies of neurons have shown high variability in mRNA expression and current densities between healthy neurons of the same type [ Schulz *et al*., 2006]. We set the minimum of our sampling range to zero (equivalent to no expression of functional ion channels) to allow our population to potentially include model neurons that produce a normal AP under normal conditions even without substantial expression of all ionic currents in the model. This can represent conditions such as a loss of function mutation that may not affect normal neuronal function but could change the response when conditions are changed, *e.g.* if another channel is blocked by a drug.

Each model was simulated under 800-ms step and 500-ms ramp protocols at its individual rheobase to match the stimulation protocols used in Davidson *et al*. [2014]. From the voltage traces from these simulations we extracted values for the eight AP biomarkers described previously: AP threshold voltage; AP peak voltage; AP slope maximum; AP slope minimum; AP full width; after-hyperpolarisation time constant; resting membrane potential; and rheobase.

We then calibrated our candidate models against experimental ranges of these AP biomarkers calculated from data in Table 3 of Davidson *et al.* [2014]. The experimental range for each AP biomarker was defined as the range spanned by the mean ±1.5 standard deviations of the data for that parameter. The choice of 1.5 standard deviations was partly arbitrary but was chosen so that the range would include approximately 90% of the total probability density of the distribution of each AP biomarker assuming a normal distribution. This was so the population would capture a large proportion of total variability in each AP biomarker while excluding extreme outliers.

### Varying conductance parameters in simulation with scaling factors

In this study, we performed simulations to investigate the effects of altering the GNav 1.8 conductance, as well as the GKdr and GKM conductances, across the whole population. In each of these simulations, we implemented this by multiplying the conductance of each model in the population by a fixed scaling factor. This mimics how drug block would affect the conductances of different neurons. For example, two neurons with GNav 1.8 values of 1 and 2 respectively subject to a scaling factor of 0.5 would have their GNav 1.8 values reduced to 0.5 and 1, respectively, equivalent to 50% Nav 1.8 channel block.

### Simulations and software

All simulations were run using NEURON version 7.5 (RRID:SCR_005393) [Carnevale, 2006], through NEURON’s Python (RRID:SCR_008394) interface. NEURON uses the adaptive time step ODE solver CVODE [ Hindmarsh *et al*., 2005] to solve model equations. All simulations were recorded at 40 kHZ (NEURON’s default) then down-sampled to 20 kHz to match the sampling rate used by Davidson *et al.* [2014]. All analysis and visualisation was performed using Python 3.7. The Python module 'drgpom' we developed to run NEURON simulations on populations of models and analyse the results is included in the extended data and the most up to date version can also be found at [ https://github.com/oliverbritton/drg-pom]. The module is also available on the Python online package repository Pypi [ https://pypi.org/] and so can be downloaded and installed with a single command. The module comes with a series of examples to guide users in how to use the module, which we hope will help potential users, who have not used populations of models before, run their own simulations.

### Visualization of balances of ionic currents during simulations using currentscapes

An advantage of computer simulations is the ability to simultaneously record the voltage trace and all individual ionic currents in each simulation. To visualise the relative contributions of all ionic currents throughout simulations we used a visualization technique called a currentscape [ Alonso and Marder, 2019]. A currentscape shows the voltage trace and the fractional contribution of each ionic current throughout a current clamp simulation. Each currentscape shows the voltage trace from a simulation at the top, followed by plots showing the fractions of each outward current in the model and each inward current in the model. This allows us to see which currents are influential at each phase of the action potential, and how this differs from model to model.

### Protocols for using and installing drgpom

This guide for installing the drgpom package for Python assumes you are installing on a Windows machine and has been tested on Windows 7 and Windows 10, and should also work on Linux (drgpom has been tested on the Fedora Linux distribution).

### Prerequisites

The use of this software requires a basic familiarity with Python. One of the best places for scientists to learn Python for scientific computing is to follow the lessons from the Software Carpentry website: https://software-carpentry.org/. It also requires installation of Python 3 and the NEURON simulation software, both of which are freely available and open source.

### Installing the dependencies for drgpom

1. Download and install the Anaconda Python 3.7 distribution ( https://www.anaconda.com/). This is a Python distribution for scientific computing including most of the key libraries needed for drgpom.
2. Install NEURON ( https://neuron.yale.edu/neuron/). NEURON is simulation software that is widely used in computational neuroscience for simulating neuronal electrophysiology. We use it to run the individual simulations and implement the models in drgpom.

**Installing drgpom and compiling models**

The simplest way to install drgpom is to download it using pip, which is a Python tool for automatically download and installing packages from online repositories. If you have installed Anaconda command window, pip will come installed with it.

You can install drgpom using pip by opening a command window (in Windows this will be a command prompt and can be opened by running the command *cmd* from the Run option in the Start Menu, in Linux this will be a terminal). From there run the command:

*pip install drgpom*


Alternatively, download the latest version from the github repository at: https://github.com/oliverbritton/drg-pom/, or the version used for this study from the repository linked in the data availability statement. To install, first unpack the download, then navigate to its directory, open a command window and run the command:

*pip install.*


Now that drgpom is installed, the ionic current model files need to be compiled by NEURON before being used. To do this in Windows, follow these instructions:

https://www.neuron.yale.edu/neuron/static/docs/nmodl/mswin.html

For Linux follow these instructions:

https://www.neuron.yale.edu/neuron/static/docs/nmodl/unix.html

In either case, the model files that need to be compiled are located in the models directory in your installed drgpom folder. If you have installed Anaconda, these will be located in your Anaconda install directory within the *Lib/site-packages/drgpom/models* subfolder.

**Using drgpom to run and analyse simulations**

Included in drgpom's examples folder is a Jupyter notebook, showing various examples of how to run simulations and save, load and view results using drgpom. Jupyter notebook ( https://jupyter.org/) is a tool that allows code, data and visualisations to be integrated together in a single interactive environment that runs in a web browser.

If you have installed Anaconda you can start Jupyter notebook either by opening a command window and typing:

*jupyter notebook*


or alternatively in Windows you can open the Anaconda folder in your start menu and click the Jupyter notebook icon in there. This will open the notebook in your default web browser, from there, navigate to the notebook of examples (examples_notebook.ipynb) in the examples folder of your drgpom installation and click to open it. A Jupyter notebook is composed of multiple cells containing either text or code. Each cells containing code can be run by selecting it and clicking the run button at the top of the screen or pressing Ctrl + Enter. All code and text in a notebook can be edited so we recommend making a copy of the notebook to experiment with.

**Running an example simulation**

To run an example simulation, open a command window in your drgpom directory (if using Anaconda this will be in the Lib/site-packages/drgpom/examples folder in your Anaconda directory) and run the script *example_simulation.py* in Python by entering the command:

*python example_simulation.py*


This should produce some output including the message “This simulation has finished running” and the results of the simulation along with simulation metadata such as model details and parameter values will be saved in a file called “example_population.pkl”. This is a pickle file, which is a format for saving Python objects so they can be reloaded. A set of images will also be saved showing the voltage traces from each model in each simulation. To see how to view results from this simulation see the example notebook.

**Configuring your own simulations using a pre-existing population of models**

1. To run a new simulation using the provided population of models, copy “example_simulation.py” and alter the options parameters to configure your simulation. The simulation parameters for this type of simulation are described in Table 1 below.
2. To run your simulation, open a command window or Jupyter notebook in the directory the simulation script is saved in.
3. If using a command window type: *python script_name.py cores=n* to run the script, where n is the number of CPU cores you want to use (or don't include cores=n to use all but one core by default). If using Jupyter notebook, run the code *%run script_name.py cores=n.* In both cases the simulation will run, leave the notebook or prompt running until it has finished. When the simulation has finished you will see a message that looks like:

**Table 1.** Parameters for a simulation using an existing population of models

Parameter	Description	Allowed values	Default value	Additional notes
pop_filename	Path to existing population of models	Any path to an existing population of models pickle file	'example_population.pkl'	
name	Name to identify this simulation	Any string	‘example_simulation'	
save_type	How to save traces from the simulation	'fig', 'trace', 'both', or 'none'	'fig'	'fig' saves the voltage traces as images, while 'trace' saves them as pickle files that include the full numerical data
save_dir	Where to save traces	A directory, or None	None	
benchmark	Whether to time individual simulations	True or False	True s	
rerun	Whether this simulation is a rerun of a previous simulation	True or False	False s	drgpom by default won't let you rerun a simulation to avoid overwriting previous results, unless you set rerun=True
outputs	Which outputs beyond voltage and time to save (only applies if saving traces)	A list of mechanism names or an empty list	[] (an empty list)	See example_save_all_ionic_currents for an example of how to use this parameter to save all ionic currents
**Simulation specific parameters are below**				Multiple simulations can be run in one script, for example a ramp and a step stimulus protocol. The parameters below need to be set for each simulation.
sim_name	Name of simulation	Any string	'ramp' and 'step'	
sim_type	Type of simulation	'iclamp' or 'vclamp'	'iclamp'	Whether to perform a current clamp or voltage clamp simulation, however voltage clamp simulations are not currently well supported so we recommend only using 'iclamp'.
amp	Amplitude of stimulus (nA)	Numeric value or None to find rheobase	None	If None is used for amp, rheobase will be searched for using progressively larger amp values will be tried until an AP is initiated or the maximum limit (5 nA) is reached
celsius	Temperature in celsius	Numeric value	32	Not currently used
delay	Delay between start of simulation and first stimulus (ms)	Positive numeric value	500	
dur	Duration of stimulus (ms)	Positive numeric value	500 for ramp, 800 for step	
interval	Interval between stimuli if num_stims > 1	Positive numeric value	0 (for 1 stimulus)	
ions	Ion types to track dynamically	An empty list or a list of ion names, currently 'Na', 'K' and 'Ca' are supported.	['Na', 'K']	
num_stims	Number of stimuli to apply	Positive integer	1	
outputs	See outputs above			
sampling_freq	Frequency to sample voltage trace for biomarker calculation and output (Hz)	Positive numeric value	20000	
stim_func	Stimulus function to use	h.IClamp or h.IRamp	h.IClamp	Used to specify whether stimulus is a step (IClamp) or ramp (IRamp) protocol
t_stop	Time to stop simulation at (ms)	Positive numeric value	1500	
v_init	Voltage to initialise simulation at (mV)	Numeric value	−65	
flags	Special flags for particular hardcoded options	An empty dictionary for a dictionary or flag name and flag value pairs (see Simulation 2 in example_simulation.py for an example).	{} (empty dictionary)	This parameter is mainly used to allow AP full width for step stimulus simulations to be computed using the threshold voltage from a corresponding ramp stimulus simulation.

“Time taken on 4 cores = 1234 seconds. Current population saved to example_population.pkl. This simulation has finished running.”

4. You can now open your population results by following the examples in the example Jupyter notebook. To open the example notebook follow the instructions in the “Using drgpom to run and analyse simulations” section above.

**Creating and calibrating a new population of models**

1. To create and calibrate a new population of models use example_population_creation.py as a template and alter the parameters in Table 1 above and Table 2 below as required. Simulation parameters are the same as those given in Table 1 for using an existing population of models.
2. Once you have created your simulation file, open a command window, navigate to the directory the file is in and enter:

*python your_simulation.py*
  where *your_simulation.py* is the name of your simulation file.
3. The simulation will now run, do not close the window before it has finished as this will halt the simulation.
4. Once the simulation is completed the results will be saved to a file that can be opened in Python. The examples notebook provides example code showing how to view your results.

**Table 2.** Parameters for creating and calibrating a population of models.

Parameter	Description	Allowed values	Default value	Additional notes
**Population Creation Parameters**				
num_models	Number of candidate models to generate	Any positive integer.	20000	This is not the final number of models in the population but the number of candidate models to be evaluated during the calibration process.
parameter_data	Names and scaling ranges for creating a new set of parameters for a population of models using Latin hypercube sampling.	Python dictionary where each in key value pair the key is the name of the parameter to be varied and the value is a list with the minimum and maximum scaling factors to be applied to that parameter in the parameter set.	{'GNav17':[0.0,0.4], 'GNav18':[0.,4.0], 'GNav19':[0.,4.], 'GKdr':[0.,4.], 'GKA':[0.,40.], 'GKM':[0.,4.], 'GH':[0.,2.], 'GKleak':[0., 0.2]}	Mutually exclusive with parameter_filename – one of these must be set to None.
parameter_filename	Filename with parameters to load.	Any allowed filename pointing to a csv file or similar where each column is a different parameter, each row is a different set of parameters and the first row of each column is the name of that parameter. An example parameter file is found in the example directory of the repository as ‘example_params.csv’.	None	Mutually exclusive with parameter_data – one of these must be set to None.
model_details	Define names of all ionic current models used in the model and the names and identities of all parameters used in the model.	Complex format, see note below.	See note below	
save_parameter_set	Whether to save the population parameter set	True or False	True	
parameter_set_filename	Filename to save parameter set to	Any valid filename	‘example_population_creation_parameters.csv’	
**Population Calibration Parameters**				
biomarkers_to_calibrate	Dictionary of biomarkers to use for calibration and which simulation to get the biomarker values from	Dictionary with format: {sim_name:[list of biomarkers], sim_name2:[list of biomarkers2], Etc.}	See definition in example_population_creation.py	
calibration_ranges	Where to get biomarker calibration ranges from	'Davidson' - Currently only calibration values from Table 3 of Davidson *et al.*, 2014, PAIN are supported.	‘Davidson’ – values from Davidson *et al.* Table 3	
std	Number of standard deviations from mean to use to define calibration ranges.	Any positive number. Recommended values are 0.5 to 2.	1.5	
verbose_calibration	Whether to print details of calibration process	True or False	False	

**Notes on the model_details parameter**

The model_details parameter defines the ion channel models that will be included within each model in the population, and which parameter(s) in each ion channel model will be varied. To define this parameter we first initialise a dictionary called model_details containing another dictionary called mechanisms that will store the details of each ion channel model:

model_details = {'mechanisms':{}}


Each ionic current model to be included in the model, and each parameter to be varied within that ion channel model is defined as follows:

model_details['mechanisms']['nrn_mech_name'] = {'param_name':'param_name_in_nrn’}


‘nrn_mech_name’ is the name of the ion channel model in NEURON. To find this name open the.mod file for the relevant current model in the Models directory of the repository and look for the line beginning SUFFIX. This gives the name of the mechanism in NEURON.

‘param_name’ is the name drg-pom will know that parameter by, for example ‘GNav 1.7’. You can choose this parameter name to be whatever you want, ideally something clear and memorable. These parameter names should correspond to those used in parameter_data to define the scaling ranges each parameter will be sampled over.

‘param_name_in_nrn’ is the name that parameter is known by in NEURON. The format of this name is the name of that parameter in the.mod file of its associated ionic current model, followed by an underscore and nrn_mech_name. For example conductances are generally given the variable name ‘gbar’, so the conductance for the kleak ion channel model is named ‘gbar_kleak’. See the *example_population_creation.py* file for an example of a complete model_details parameter.

**Running a simulation with multiple channel block conditions on multiple channels**

The simulation script *vary_amp_and_gnav18.py* in the examples directory provides an example of a simulation design similar to those used in the paper where multiple simulations are run simulating changes in multiple conductances at multiple different levels. This example can be used as a template to design your own simulation studies.

## Results

### Properties of the population of human DRG neuron AP models

The population of hDRG neuron AP models consisted of 328 models each of which had the same underlying equations but a different combination of eight ionic conductances. All models in the population had substantially different combinations of conductances, but all of them produced AP biomarkers at rheobase that were within 1.5 standard deviations of the mean values of each parameter, based on data recorded from *ex vivo* hDRG neurons [ Davidson et al., 2014].

**Figure 1.**
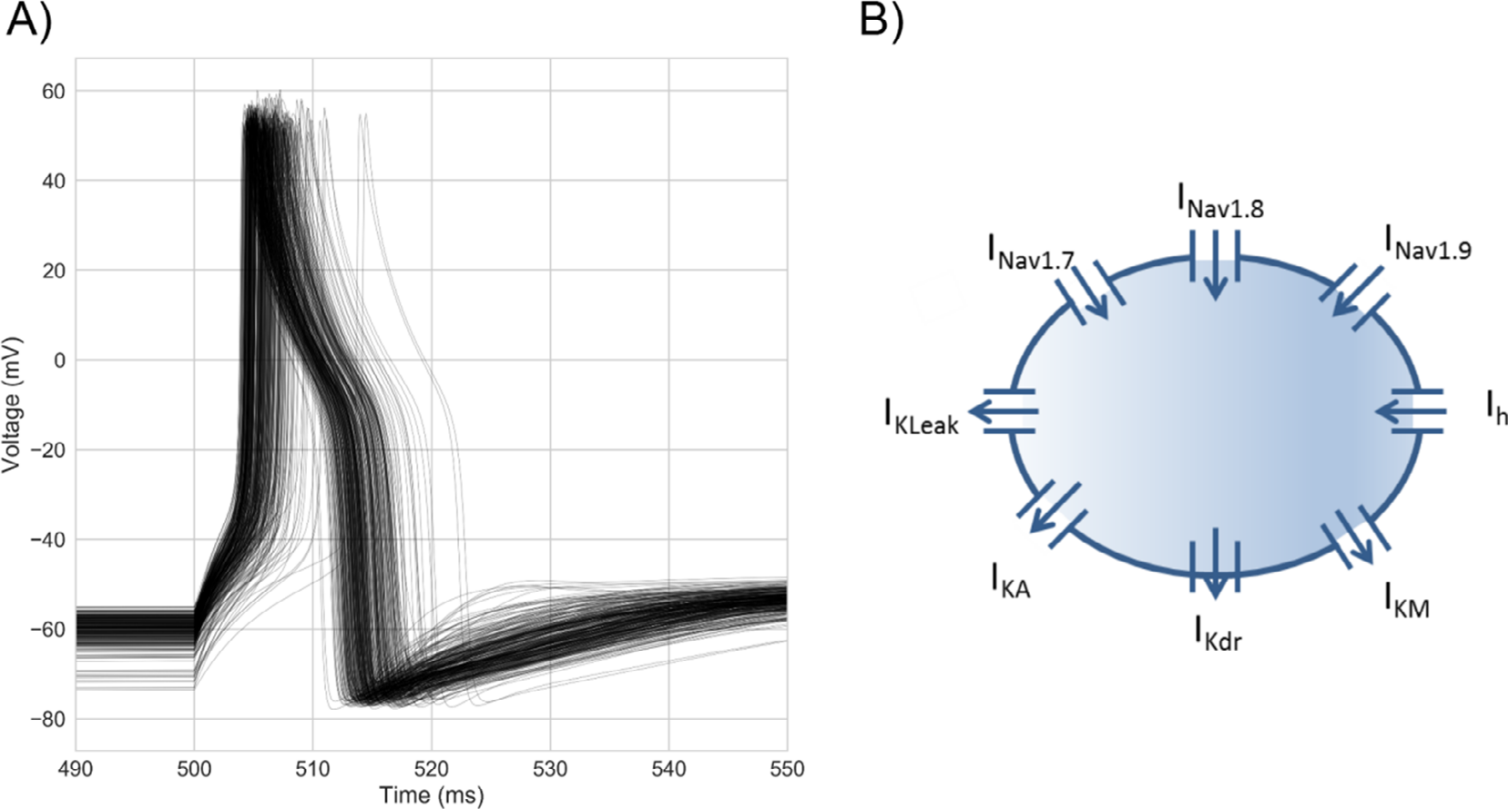
Experimentally-calibrated population of 328 human DRG neuron AP models. A) Overlaid voltage traces from every model in the population stimulated with a step stimulus at each model’s rheobase. Each model was calibrated against hDRG neuron AP biomarker ranges calculated from data in Davidson *et al.* [2014]. B) Model schematic. The model approximates the hDRG neuron soma as a single compartment Hodgkin-Huxley model with 8 ionic currents. Arrows indicate the usual direction of current flow for each current. Each model in the population has the same 8 ionic currents but different channel densities of each to simulate inter-neuronal variability in ionic conductances.

Voltages traces from the population of models ( Figure 1A) show they reproduce key characteristics of the hDRG neuron AP, including its characteristic broad shoulder [ Davidson *et al.* 2014, Han et al., 2015], the slow rise to threshold followed by a rapid upstroke, and the presence of an after-hyperpolarization following repolarization. A schematic of the baseline model is shown in Figure 1B.

**Figure 2.**
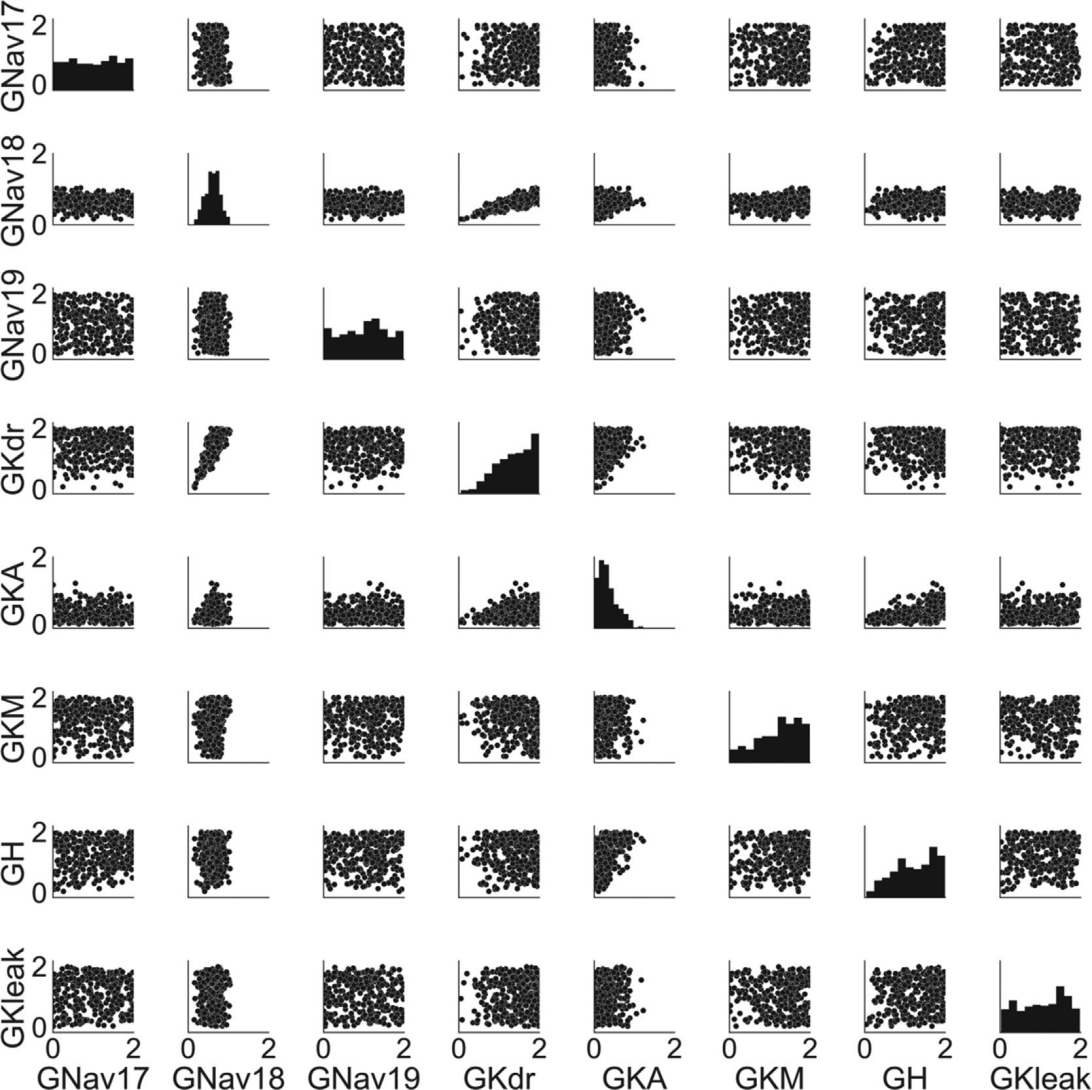
Scatter plots and histograms of the distribution of conductances in the population. Histograms on the diagonal panels show the distributions of individual conductances. Scatter plots show distributions for all pairs of conductances. Conductances are given in terms of a dimensionless scaling factor applied to the baseline conductance values (which are provided in the supplementary material).

The traces in Figure 1A were produced by models with a diverse range of conductance profiles ( Figure 2). Most conductances (except for GNav 1.8 and GKA) span the sampled conductance range. For GNav 1.8 and GKA, the accepted models only have a subset of the sampled conductance range (GNav 1.8: 0.15 – 1.04, GKA: 0 – 1.22). For GNav 1.8, this is because models with low GNav 1.8 have peak voltages that are below the experimental range, and models with high GNav 1.8 have threshold voltages that are below the experimental range. For GKA, no model with high GKA had a combination of threshold voltage, peak voltage, and after-hyperpolarisation time constant that was simultaneously within the experimental ranges of these three AP biomarkers.

Most of the pairs of conductances are uncorrelated ( Figure 2), however GNav 1.8 and GKdr have a strong positive correlation (r = 0.82). This correlation is caused by calibrating on a combination of three biomarkers: threshold voltage, peak voltage and AP full width parameters, as removing all of these AP biomarkers from the calibration process also removes the correlation between GNav 1.8 and GKdr and removing any one of them reduces the correlation coefficient. This indicates that in our model the balance of these two currents is important throughout the action potential, from upstroke (threshold, peak voltage) to repolarisation (AP full width).

### Visualising the balance of currents in different hDRG models

**Figure 3.**
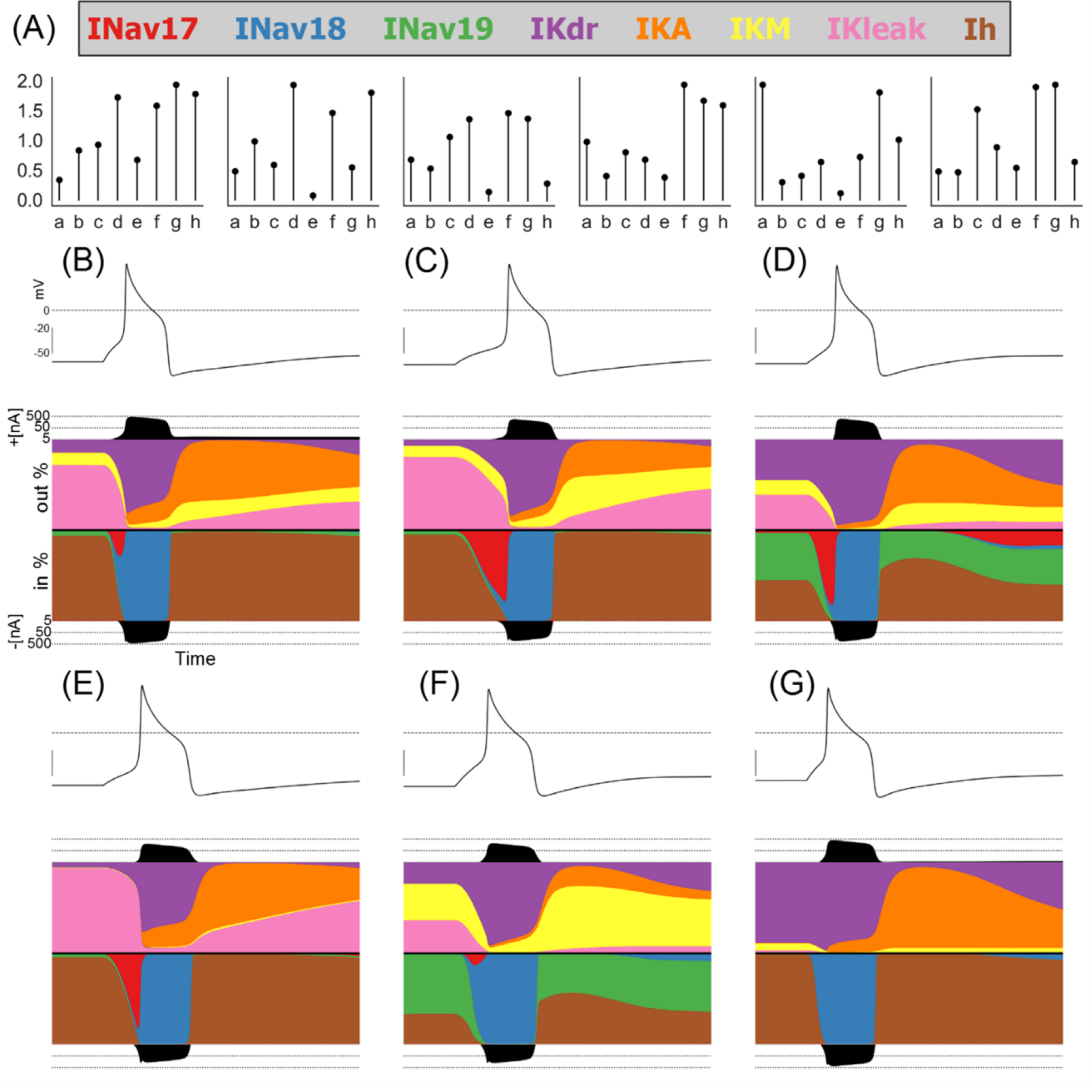
Conductances and ionic currentscapes for six different models from the population of models. A: Conductance scaling factors for six models selected to show a wide variety of different conductances and ionic current profiles. B-G: Currentscapes [ Alonso and Marder, 2019] showing the fractions of each ionic current active during a simulated step stimulus. Each plot shows, from top to bottom, the voltage trace, the total outward current, the fraction each outward current contributes to the total outward current during the simulation, the fraction each inward current contributes to the total inward current during the simulation, and the total inward current.

As can be seen in Figure 2, we found many different combinations of conductances that produced viable models consistent with observed experimental variability in hDRG AP biomarkers. However, while each of these models produced similar voltage traces, the different conductances in each model mean that the balance of underlying ionic currents in each model were substantially different. Figure 3 shows the conductances and relative magnitudes of each current in six models from the population using a visualisation technique called a currentscape [ Alonso and Marder, 2019] (see Methods for details).

We see that during quiescence the primary active currents are IKdr (purple) and IKleak (pink) for the outward currents and Ih (brown) and INav 1.9 (green) for the inward currents. The relative proportions of these currents are however highly variable from model to model. Similarly, the main currents during the upstroke are INav 1.7 (red) and INav 1.8 (blue), but some models have a large contribution by Nav 1.7 and in some models the contribution is very small, while the contribution of INav 1.8 is uniformly large.

During the body of the AP, in all models IKdr (purple) is the dominant outward current and INav 1.8 (blue) is the dominant inward current. These two currents appear to have unique roles in shaping the AP that cannot be performed by any of the other currents in our model. This can explain why GKdr and GNav 1.8 are highly correlated in Figure 2; the values of both conductances must be balanced against one another as no other currents can perform their roles in shaping the AP.

Following repolarisation, the potassium currents IKA (orange) and IKM (yellow) are both important for shaping the after-hyperpolarization. However, the balance of these two currents differs from model to model. For example, the model in Figure 3E has most of its post-repolarisation outward current conducted through IKA with a very small contribution from IKM, while the model in Figure 3F relies primarily on IKM with a small contribution by IKA. As we show later, the balance of these currents is not as critical as the balance of GNav 1.8 and GKdr at rheobase, where only a single AP is fired. However, at higher stimulus amplitudes that support repetitive firing, the value of GKM becomes important for modulating firing rate.

### The Nav 1.8 conductance has non-linear effects on firing rate

**Figure 4.**
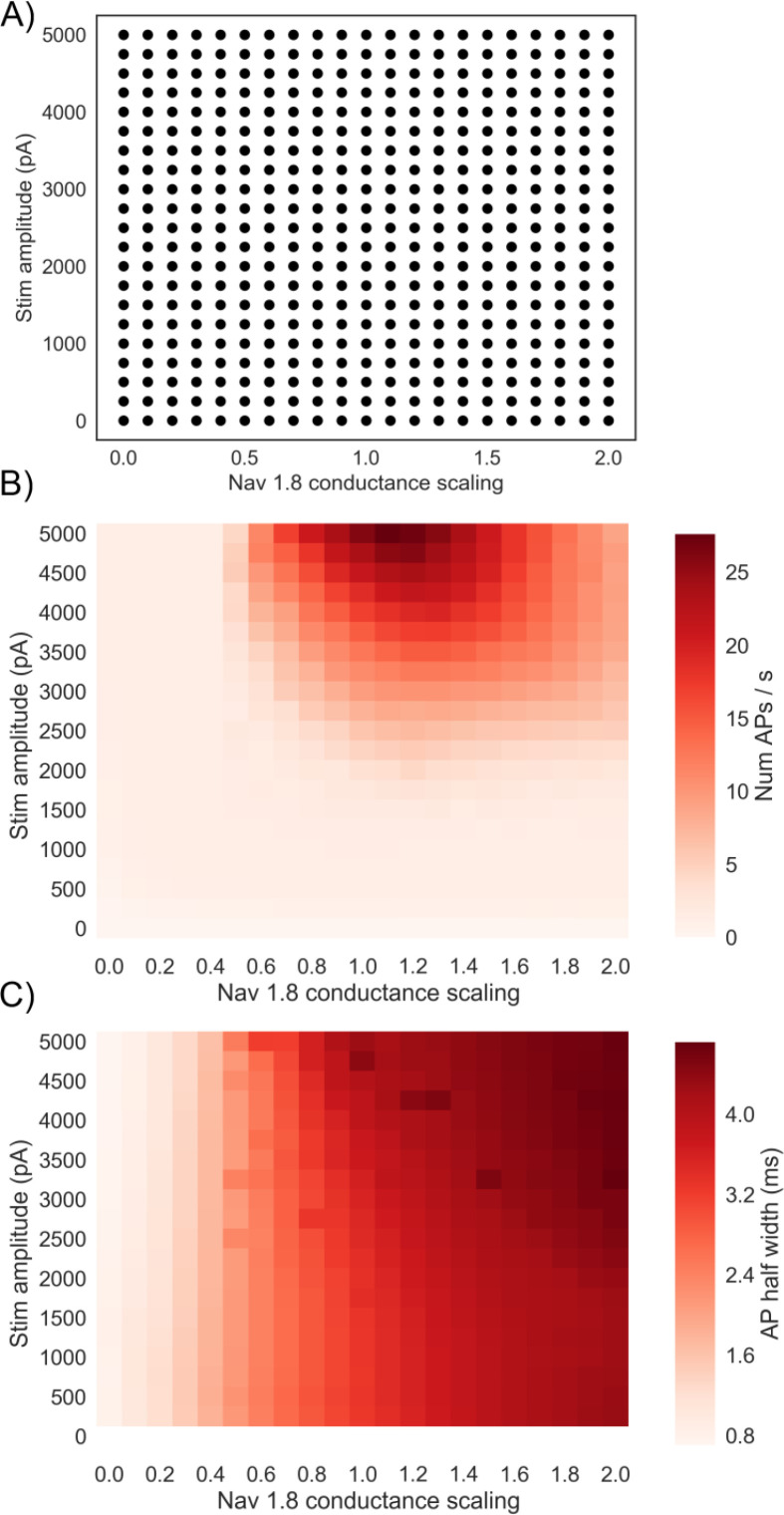
Effects of co-varying Nav 1.8 conductance and stimulus amplitude on firing rate and AP half width. A) Simulation study design showing the grid search design used over stimulus amplitude and GNav1.8. Each dot represents a simulation on all 328 models in the population with the given Nav 1.8 conductance scaling factor applied to all models, and an 800 ms step stimulus current applied with the given amplitude. B) Mean firing rate of the population for each of the different simulation conditions. Each square represents a simulation of all models in the population with a different pair of values for GNav 1.8 scaling factor and stimulus amplitude. C) Mean AP half width of the population for each of the different simulation conditions.

Following the evaluation of the population of hDRG neuron models shown in the previous section, we investigated how changing the Nav 1.8 conductance affects hDRG neuron excitability across a wide range of different backgrounds of other ionic conductances. We therefore simulated different levels of block and enhancement of the Nav 1.8 conductance in every model in the population, across a range of stimulus amplitudes. Figure 4A shows the GNav 1.8 scaling factor and stimulus amplitude parameters for the 441 simulations that we ran. For each simulation the 328 models in the population were each simulated under an 800 ms step stimulus current protocol. The Nav 1.8 conductance was varied by multiplying GNav 1.8 in each model by a scaling factor, which is similar to how drug block or enhancement would affect the conductance.

Figure 4B shows the mean firing rate (APs/s) of the population of models in each simulation. Mean firing rate is not maximised at the highest GNav 1.8 we simulated but is instead maximised when GNav 1.8 is slightly above the baseline value (between 1.1 and 1.3 times baseline, depending on the stimulus amplitude of the simulation). If GNav 1.8 is increased beyond this point, the mean firing rate decreases. This result implies that increased expression of Nav 1.8 could lead to a decrease in firing rate. We also find that reducing GNav 1.8 to 0.5 times baseline or less is sufficient to stop repetitive firing at all of the stimulus amplitudes we simulated, indicating the importance of Nav 1.8 in our model for enabling repetitive firing.

We hypothesised that one reason for the decrease in firing rate as GNav 1.8 is increased could be increased AP width due to increased Nav 1.8 current during AP repolarisation. A wider AP could reduce the maximum firing rate of a neuron by lengthening the minimum time between subsequent APs. Unlike other sodium channels Nav 1.8 is active during AP repolarisation, contributes to the characteristic shoulder of the DRG action potential [ Han et al., 2015], and is therefore important for determining AP duration. Figure 4C shows that mean AP half width (defined as the period during an AP in which voltage is continuously above a threshold halfway between the threshold voltage and peak voltage) increases steadily with increased GNav 1.8 and is insensitive to stimulus amplitude. For example, averaging over different stimulus amplitudes, mean half-width of the population changed from 3.6 ms at baseline (GNav 1.8 = 1) to 4.5 ms when GNav 1.8 is doubled (GNav 1.8 = 2).

**Figure 5.**
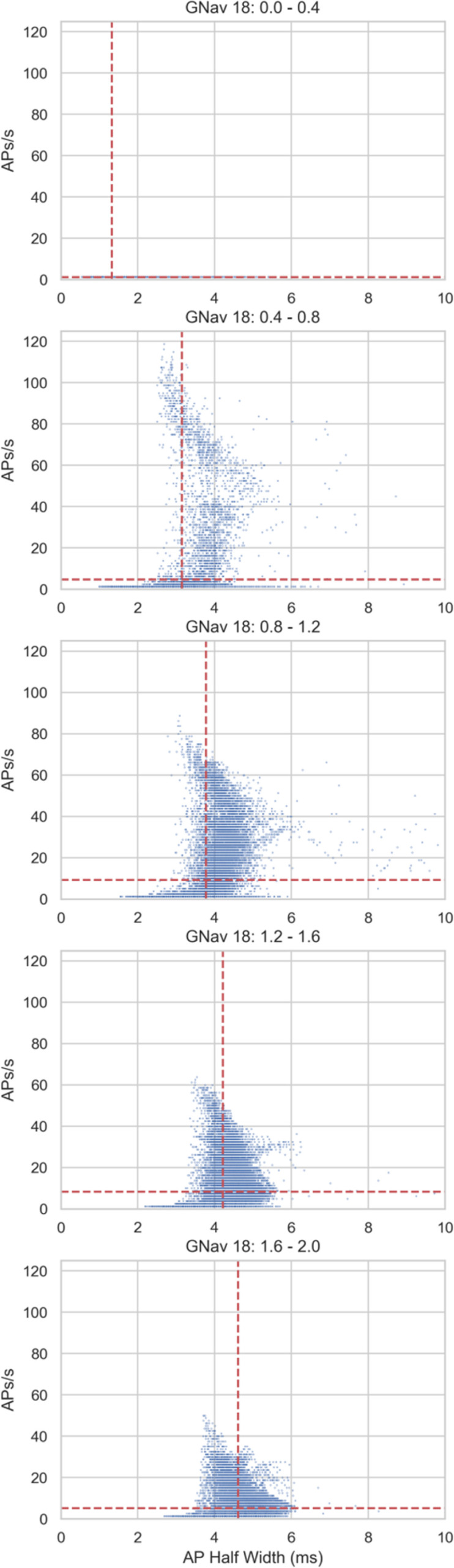
Relationship between AP half width and firing rate for individual models in the population of models under different GNav 1.8 scaling factors. Blue dots show results from individual models in different simulations from the simulation design shown in Figure 4A. Red lines show mean values of plotted results. Descending panels show results from simulations with progressively larger GNav 1.8 scaling factors.

Plotting the relationship between half width and firing rate in individual models ( Figure 5) shows that firing rates within the physiological range (approximately 0 – 25 AP/s) can occur across a range of half-widths, but the very high firing rates that some models in the population exhibit (up to approximately 120 AP/s) only occur at the lower half-widths found predominantly in simulations with lower GNav 1.8 scaling values (e.g. Figure 5, second panel from the top). However, at the lowest values of GNav 1.8, there is very little repetitive firing ( Figure 5, top panel). Therefore, one of the effects of Nav 1.8 on hDRG neuron electrophysiology may be to support repetitive firing but also limit the maximum firing rate by broadening the AP.

### Very high firing rate models are distinguished from other models by low GKM and high GKDr potassium conductance

We noticed that a substantial fraction of models fired at a very high rate (> 20 AP/s) ( Figure 5. This is above the physiological values reported from real hDRG neurons [ Meyer and Campbell, 1981] but these models are still relevant because they exhibit a normal hDRG-like AP at rheobase. The ways that these models’ conductance profiles differ from models in the population that do not fire at a very high rate could indicate which currents act as brakes or enhancers of excitability when the Nav 1.8 conductance is altered.

**Figure 6.**
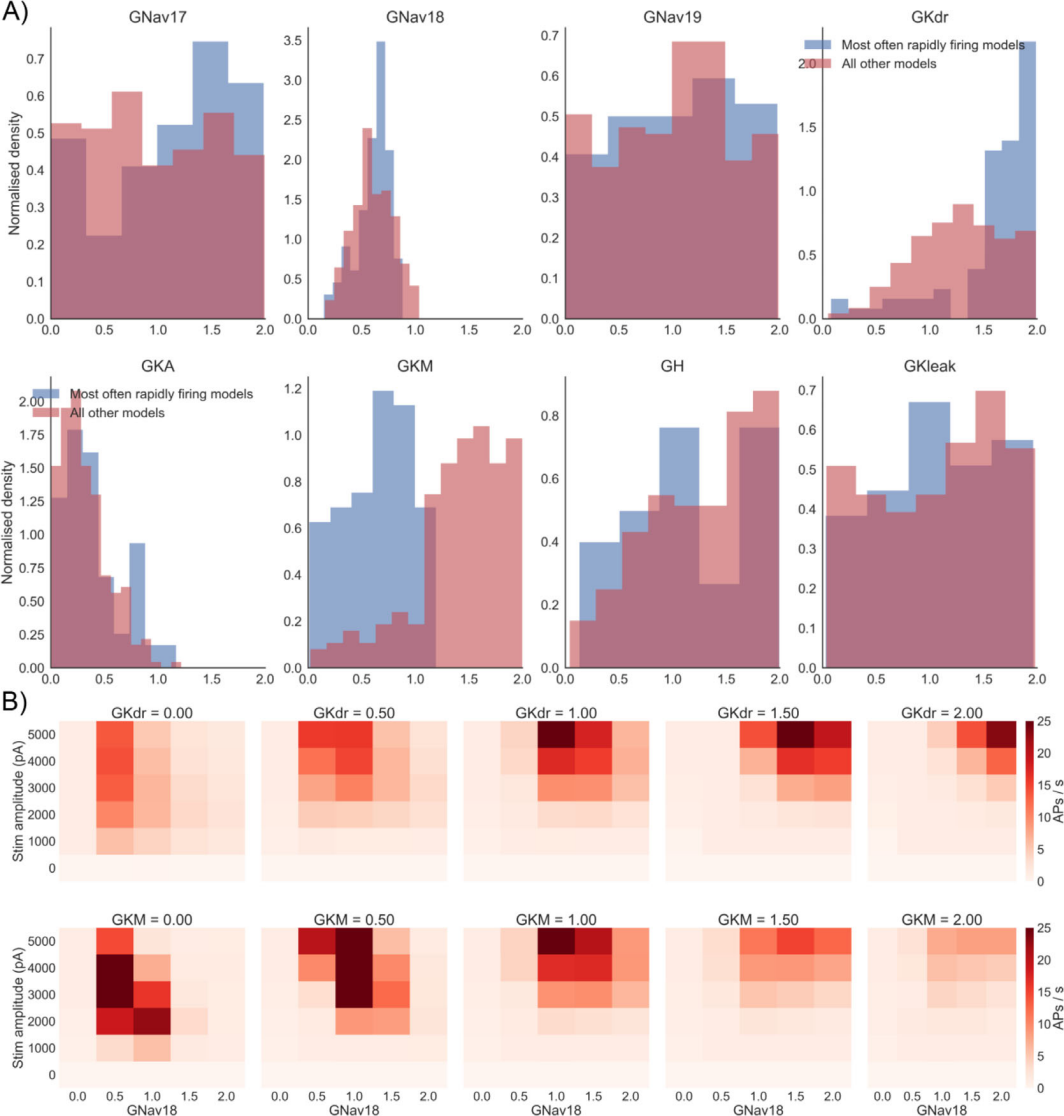
GKdr and GKM are key determinants for whether a model fires rapidly under many different combinations of GNav 1.8 and stimulus amplitude. A) Conductance distributions of two subpopulations in the population of models. Blue histograms show conductance distribution for the top 25% of models ranked by the number of simulations in Figure 4 in which they fired at a very rapid rate (> 20 AP/s). Red histograms show the other 75% of models. Histograms of the two subpopulations are normalised as the number of models in each subpopulation were not equal. B) Results of varying GNav 1.8, stimulus amplitude and one of GKdr (top row) or GKM (bottom row). Each square in a plot shows the mean firing rate across the population of models for one simulation.

Therefore we divided the population of models into two groups based on the number of simulations from Figure 4A in which a model fired at a rate > 20 AP/s. The top 25% of models were assigned to the “most often rapidly firing” group” and the other 75% of models to the “all other models” group. Figure 6A shows the distribution of each conductance in each group. Interestingly, GKdr and GKM differ substantially between the two groups. GKM is much smaller in the most often rapidly firing group (0.64 ± 0.32) than the other models group (1.41 ± 0.43). GKdr is larger in the rapidly firing group (1.60 ± 0.44) compared to the other models group (1.28 ± 0.43). That GKdr is actually larger in the rapidly firing group suggests it is not just a lack of total potassium current conductance, and a corresponding lack of outward current to oppose repetitive firing, that causes these models to fire so rapidly.

**Figure 7.**
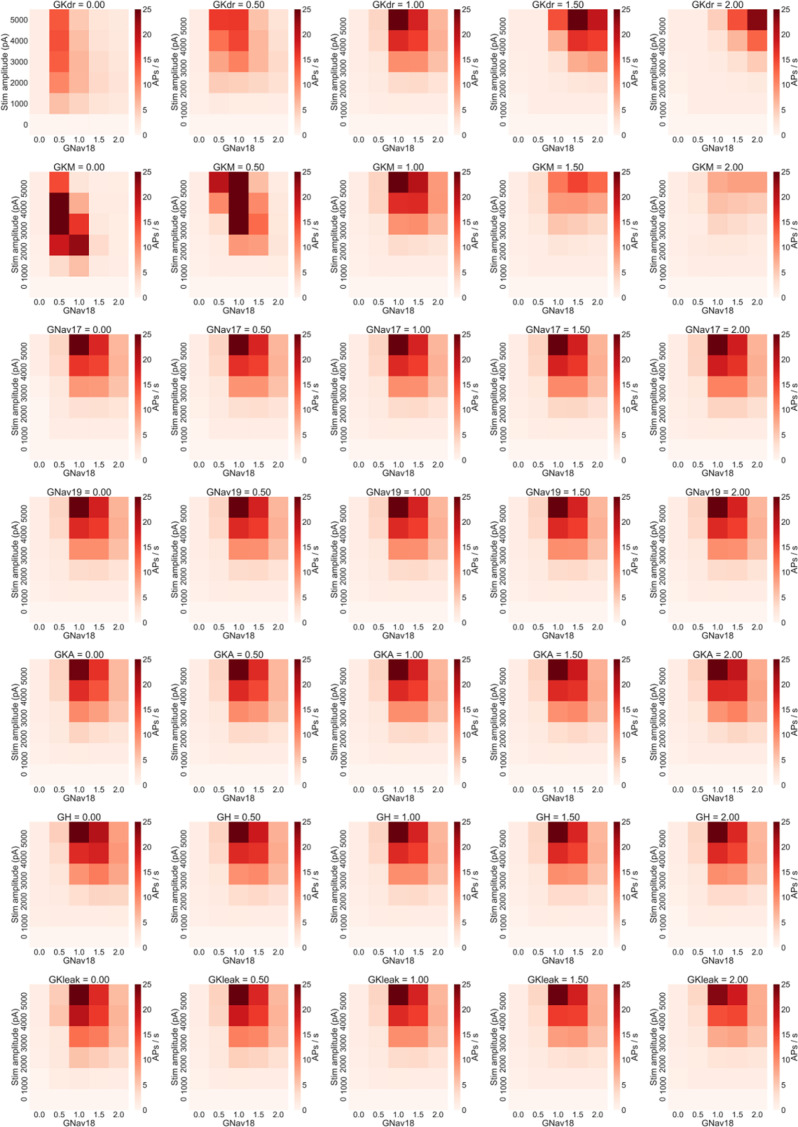
Average firing rate of the population of models when non-Nav 1.8 conductances are varied. Each row of plots shows the results of changing one non-Nav 1.8 conductance to 0%, 50%, 100%, 150% and 200% of each model in the population’s original value and rerunning the simulation study design shown in Figure 4A. Only variation in GKdr and GKM show substantial effects on firing rate. Within each plot each square represents the mean number of AP/s fired during a step stimulus averaged across all models in the population.

We therefore simulated the effects of modulating each combination of Nav 1.8 and one of the other seven conductances in the model to confirm whether the firing rate of models in the population were sensitive to GKdr and GKM, but no other conductances. Modulating either GKdr or GKM substantially altered the effects of varying GNav 1.8 on firing rate ( Figure 6B), while the other five conductances showed little effect on firing rate ( Figure 7). Modulating GKdr ( Figure 6B, top row) altered firing frequency by increasing the Nav 1.8 values required for high frequency repetitive firing as GKdr was increased. Modulating GKM primarily reduced firing rate across all GNav 1.8 values as GKM was increased.

### M-type and delayed-rectifier potassium currents modulate the effects of Nav 1.8 and collectively control excitability

As we found that GKM and GKdr levels each have an important role in determining the excitability of models in the population ( Figure 6B), we ran a simulation study where we co-varied four parameters: stimulus amplitude; GNav 1.8; GKdr; and GKM, to see how changes in these three important conductances interacted.

As we were simulating changes in four parameters, we used a coarser range of values for each parameter compared to the previous simulation study design ( Figure 4A). Stimulus amplitude was varied from 0 to 6000 pA in increments of 1000 pA and the conductance scaling factors for GNav 1.8, GKdr and GKM were each varied from 0.0 to 2.0 in increments of 0.5. All combinations of the four parameters over these ranges were simulated for a total of 875 simulations on each model in the population.

**Figure 8.**
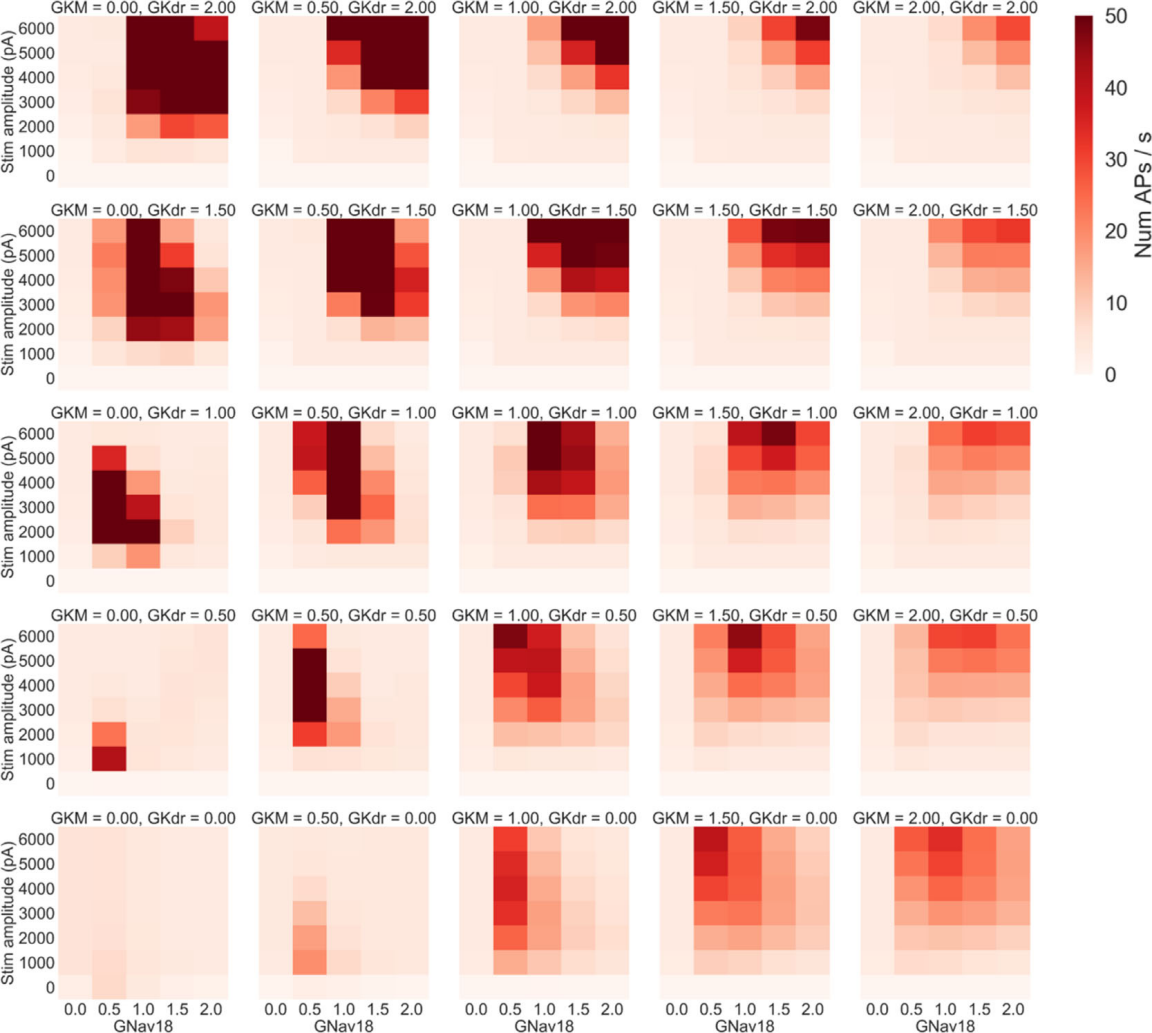
Average firing rate of the population of models when GKM, GKdr, GNav 1.8 and stimulus amplitude are co-varied. Each panel shows the results of simulations with a specific pair of GKM and GKdr scaling factors applied. Within each panel each square represents the mean number of AP/s fired during a step stimulus averaged across all models in the population with GKM, GKdr and GNav1.8 scaled by the relevant values. GKM increases in each panel from left to right, while GKdr increases from bottom to top.

Figure 8 shows the mean firing rate of the population in each of these simulations. As plots go from left to right, GKM increases, as plots go from bottom to top, GKdr increases.

Four main trends can be seen in Figure 8:

1. Increasing GKM generally decreases firing rate as can be seen by comparing the rightmost column (GKM = 2) to the central column (GKM = 1).
2. Increasing GKdr increases firing rates at higher GNav 1.8 values but decreases firing rate at lower GNav 1.8 values – as can be seen by noting how in each column going from the bottom (GKdr = 0) to top (GKdr = 2) of that column the peak firing rate in each panel moves to the right (to higher GNav 1.8 values).
3. Decreasing GKdr and GKM to very low levels almost complete prevents rapid firing – in the bottom left corner of the figure the mean firing rate in most simulations is close to 2 AP/s. This is primarily due to most models failing to repolarise. For example in the set of simulations where GKM and GKdr were set to 0 (bottom left panel, Figure 8), in every simulation where neither GNav 1.8 nor stimulus amplitude were set to 0 every model in the population exhibited repolarisation failure.
4. When the sum of GKdr and GKM is low, decreasing GNav 1.8 from baseline can cause mean firing rate to decrease. This can be seen in many of the panels in the bottom left quadrant of Figure 8, for example in the case where GKdr = GKM = 0.5, decreasing Nav 1.8 by 50% causes a dramatic increase in firing rate at almost all tested stimulus amplitudes.

**Figure 9.**
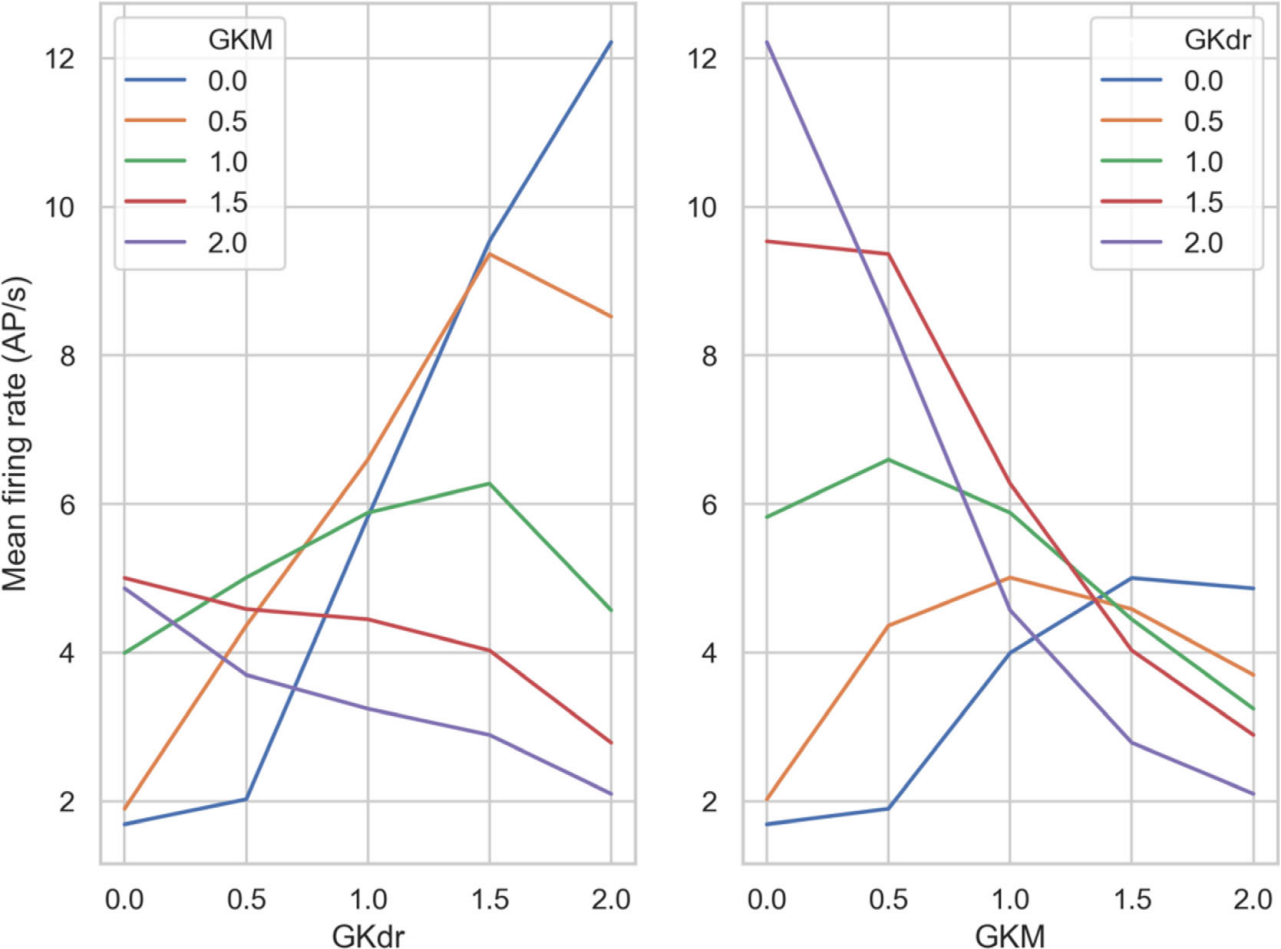
Mean firing rate of the population of models averaged over all scaling factors of GNav 1.8 simulated in Figure 8. Left) Each line shows a different GKM scaling factor, with GKdr scaling factor varying on the x-axis. Right) Each line shows a different GKdr scaling factor, with GKM scaling factor varying on the x-axis.

To better understand the relationship between the values of GKdr and GKM and their effects on firing rate, we looked at the overall trends for these conductances. We averaged mean firing rate over all of the GNav 1.8 scaling values we simulated. This condenses the information in each panel of Figure 8 down to a single averaged firing rate. We then plotted curves of either GKM or GKdr against mean firing rate averaged over GNav 1.8. This produced a group of curves ( Figure 9) that show the general trend from varying GKdr ( Figure 9A) or GKM ( Figure 9B).

Broadly we see three different relationships between firing rate and the scaling factor of GKdr and GKM, depending on the scaling factor of the other conductance:

1. Monotonically negative response - increasing the potassium conductance that wasn’t fixed always decreased firing rate – the expected response for a potassium current. This occurred when GKM or GKdr scaling factors were fixed at 1.5 and 2 ( Figure 9A and B, red and purple curves).
2. Monotonically positive response - increasing the potassium conductance that wasn’t fixed always increased firing rate. This is opposite to the expected response for a potassium conductance and occurs because without sufficient potassium current the models cannot effectively repolarize, which prevents repetitive firing. This only occurred when GKM was fixed at 0.
3. Inflected response – increasing the potassium conductance that wasn’t fixed increased firing rate up to an inflection point, beyond which increasing the conductance further caused firing rate to decrease. This is the most common scenario.

These results shows that in our model, across a wide range of ionic conductance profiles, the response of firing rate to a change in a potassium conductance can qualitatively differ depending on the value of another potassium conductance. Therefore channel block by a drug could increase or decrease firing rate, depending on the ionic background of the hDRG neuron the drug was applied to.

## Discussion

### Main findings

In this study, we investigated the roles the voltage-gated sodium channel Nav 1.8 and the IKdr and IKM potassium currents play in determining hDRG neuron excitability using a population of computational models calibrated against experimentally determined hDRG AP biomarker ranges. We used this population of models methodology to allow us to simulate the variability in ion channel densities found in real neurons and investigate how variability in many different ionic currents collectively interact to determine overall cellular excitability.

The main findings of this study were:

1. We developed a new model of the hDRG neuron AP using published data from hDRG neurons at the AP level and at the ion channel level where data was available. We used this model as a baseline to develop a population of models that integrated experimentally observed variability in hDRG AP biomarkers.
2. Altering the Nav 1.8 conductance had nonlinear effects on excitability. Decreasing the Nav 1.8 conductance from baseline in each model decreased the mean firing rate of the population, however increasing the Nav 1.8 conductance initially increased firing rate up to a point, beyond which firing rate decreased.
3. Simulation studies with the population of models identified key roles for the M-type potassium current and delayed-rectifier potassium current. Increasing the M-type potassium current conductance decreased firing rate. However, increasing the delayed-rectifier potassium current increased firing rate, but only when Nav 1.8 was also increased by a similar factor.
4. Several of our results agree with the literature on DRG neuron electrophysiology, which provides credibility for our modelling approach. Increasing Nav 1.8 conductance increased AP width, in line with current understanding of the role of human Nav 1.8. [ Han et al., 2015]. Our finding that increasing M-type potassium channel conductance decreased excitability agrees with studies that show M-channel opening drugs such as retigabine reduce sensitivity to nociceptive stimuli in peripheral nerve fibres [ Passmore *et al.* 2012, Vetter *et al.* 2013, Huang *et al.* 2016].

### The hDRG neuron model

The baseline hDRG model we developed has several novel features that make it adapted to modelling hDRG neurons. We used ionic current models developed from recordings of human channels, for Nav 1.7, Nav 1.8 and Nav 1.9 [ Vasylyev et al., 2014; Han et al., 2015; Huang et al., 2014]. It was particularly important to have a human-specific Nav 1.8 model as it has substantial differences in kinetics compared to rat Nav 1.8 [ Han et al., 2015]. The kinetics of inactivation of human Nav 1.8 are slower, and the V1/2 of inactivation is more depolarized, so more human Nav 1.8 channels are available, for longer, during the action potential, compared to rat Nav 1.8. Previous models and most studies of DRG neuron electrophysiology have used rodent DRG neurons due to the difficulty of acquiring hDRG neurons for research. Therefore, species differences in AP morphology and ion channel behaviours [ Han *et al.* 2015] were not captured.

To mimic inter-neuronal variability in ion channel conductances and AP biomarkers we used the population of models methodology [ Britton et al., 2013] and experimental data from *ex vivo* hDRG AP recordings to calibrate the population of models to match the ranges of AP biomarkers from those experiments. Similar approaches to this have been used in many modelling studies of other neuron types (e.g. [ Prinz et al., 2003, 2004]) and of cardiac cells (e.g. [ Britton *et al.* 2013, Passini et al., 2017, Muszkiewicz et al., 2016]) but to our knowledge they have not previously been used in studies of DRG neurons.

Where hDRG neuron-specific data was not available we built on previous modelling studies of DRG neurons, in particular work by Tigerholm *et al.* (2015) and Choi and Waxman (2011). Tigerholm *et al.* investigated activity-dependent slowing in a model of a DRG neuron axon. As human-specific potassium current and hyperpolarisation-activated cation current models were not available we used the same potassium and h-current models that were used in this study. Choi and Waxman investigated how varying GNav 1.7 and GNav 1.8 affected excitability in a single compartment model of a DRG neuron cell body. We adapted the geometry and passive membrane properties used in this study for our model.

### Implications for the 3Rs (reduction, refinement and replacement) of animals in research

Computational hDRG neuron models, along with other alternatives such as *ex-vivo* [ Davidson et al., 2014] and stem cell-derived hDRG neurons, offer alternatives that could replace the use of rodent DRG neuron recordings in the future. For example, a computational model could be used to investigate how different ion channel modulating drugs interacted with a pathological ion channel mutation in hDRG neurons, to determine if they could reduce or counteract the pathological effects of the mutation. This type of study would require a large number of rodent DRG neurons if performed using an animal model, as the experiment would need to be repeated for each different drug tested. Computational models also have the advantage that they can be refined and iterated upon as new data becomes available, to make the most of the limited availability of hDRG neurons and recordings. Furthermore the fact that they are human-based allows for easier clinical translation, as animal moedels present patho-physiological differences with human cells, which can impact therapy outcomes.

To estimate the number of animals currently used in studies of rodent DRG neuron excitability, that could potentially be replaced by these alternatives, we searched PubMed to estimate the use of rodents in this class of experiment. Our base search query was: (mouse OR mice OR rat OR rodent) AND (nociceptor OR DRG OR dorsal root ganglion) AND pain AND (ion channel OR ion channels OR Nav) with the addition of:

1) AND (drug block OR channel block OR pharmacological block OR current inhibition OR current block)
2) AND (mutation OR channelopathy)

These queries aimed to find papers that investigated: 1) Drug-induced changes in human DRG excitability and 2) Changes in human DRG excitability caused by ion channel mutations, as these are the main areas that could be investigated with our population of models.

The base search produced 1018 papers published over the last 5 years (2015-20), indicating wide use of these models. Filtering further using Queries 1 and 2 reduced these papers to 361 unique non-review papers published between 2015 and 2020. We analysed the first 20 papers for each query to determine average numbers of animals used for DRG studies only. Mean animals used for DRG studies in the 16/40 papers where number of animals used was reported or could be inferred from figures was 103, with a range of 24 to 273. Therefore, annual animal use in these experiments is estimated at 7,400 animals (103 animals multiplier by 361 papers divided by 5 years), while total annual animal use in all DRG experiments is estimated at 21,000 animals (using the figure of 1018 papers found by the base query). These estimates do not include animal use in industry, which is likely to be substantial given the current interest in developing selective sodium channel blockers targeting human DRGs.

### Nav 1.8 and GKdr

Changes to ionic currents rarely affect only a single channel. For example, pathological changes in channel expression often affect multiple channels [ Black et al., 2004; Cao et al., 2010; Zhao et al., 2013], and even selective ion channel blocking drugs often have non-negligible side effects.

Nav 1.8 is known to support repetitive firing in DRG neurons [ Renganathan et al., 2001; Blair and Bean, 2002], due to its rapid recovery from inactivation [ Dib-Hajj et al., 1997]. Therefore we expected that increasing GNav 1.8 would likely increase firing rate across the population. However, our simulations showed a more complex relationship. Increasing GNav 1.8 by up to approximately 30% increased mean population firing rate but beyond this inflection point increasing GNav 1.8 caused the mean population firing rate to decrease. We also found that the GNav 1.8 value at which this inflection point occurred was highly dependent on GKdr ( Figure 9B). Enhancing or blocking GKdr caused the inflection point to move to higher or lower GNav 1.8 values respectively.

The opposing contributions of Nav 1.8 and IKdr during repolarisation of the action potential ( Figure 3) suggest the balance between Nav 1.8 and IKdr may be important for supporting repetitive firing in hDRG neurons. However, as more ionic currents are expressed in real hDRG neurons than are included in our model, it is likely the true relationship will be more complicated than simply balancing GKdr against GNav 1.8.

### M-type potassium current

We found that the conductance of the M-type potassium current, primarily carried by Kv 7.2, 7.3 and 7.5 in DRG neurons [ Passmore et al., 2003], strongly modulated the excitability of the population of models. Increased GKM decreased mean firing rate of the population of models across a wide range of stimulus amplitudes and GNav 1.8 and GKdr scaling factors ( Figure 8). This is in agreement with studies showing that M-current activators such as retigabine and flupirtine can reduce sensitivity of nociceptors to nociceptive stimuli [ Passmore et al., 2012; Vetter et al., 2013; Huang et al., 2016]. The accepted role of the M-current is to stabilize the membrane potential against small depolarisations. Our results suggest that the M-current could also have a role in opposing large stimulus currents during sustained firing, due to the M-current’s slow kinetics of activation that lead to it reaching its maximum amplitude only after a period of repetitive firing ( Figure 3).

### Future development and limitations

We think that future development of the model presented here should focus on three areas: validation against new experimental data as they become available; incorporating additional ionic currents, particularly calcium-activated potassium currents and calcium currents; and expanding the geometry of the model to include the DRG neuron axon and t-junction with the cell body.

The population of models used in this study was calibrated using AP biomarker data from wild type hDRG neurons stimulated under standard conditions (e.g. no applied drugs). Adding additional calibration steps with AP biomarkers from experiments under very different conditions (e.g. under drug block) would further constrain the allowed conductance profiles of accepted models which should result in a population that behaves more realistically under a wider range of simulated conditions. This overcomes an important limitation from previous models, which are based in animal data.

Our current baseline model contains eight ionic current models that cover a diverse range of sodium and potassium currents but do not include calcium currents, calcium-activated potassium currents, or ionic pumps and exchangers. All of these would be good targets to add to the model however there is less data, particularly DRG-specific data, to parameterise models of these currents, so this work would either require using models developed for other neuron types or new experimental data.

Extending the population of models to include a realistic DRG neuron geometry would allow new research questions to be considered and could extend calibration of the population, for example models could be calibrated against the known range of conduction velocities in C-fibres. The variability in conductance could also be extended to account for differences in ion channel expression at different parts of the axon and cell body [ Calvo et al., 2016; Tsantoulas and McMahon, 2014].

## Data availability

Zenodo: Data supporting “Changes in human dorsal root ganglion neuron excitability from modulating Nav 1.8 conductance are non-linear and depend on the conductances of the delayed rectifier and M-type potassium currents: a simulation study”. https://doi.org/10.5281/zenodo.5512414

This project contains the following underlying data:

• drg-pom-master.zip (Source code, models and examples for this study and drgpom software.)
• Figure_1_data.csv (Voltage trace data underlying Figure 1A.)
• Figure_2_data.csv (Conductance scaling factors for each model in the population of models, underlying Figure 2.)
• Figure_3A_data.csv (Conductance scaling factors for the models in Figure 3.)
• Figure_3B_model_[1/2/3/4/5/6]_data.csv (Voltage and current traces underlying Figure 3B.)
• Figure_4A_data.csv (Simulation parameter data underlying Figure 4A.)
• Figure_4BC_data.csv (Mean number of APs; AP half width data; and simulation parameters underlying Figure 4B and 4C.)
• Figure_5_data.csv (Half width and AP firing rate data for individual models underlying Figure 5.)
• Figure_6A_data.csv (Conductance and model category data underlying Figure 6A.)
• Figure_6B_GKdr.csv (Mean number of APs and simulation parameters underlying the top row of Figure 6B.)
• Figure_6B_GKM.csv (Mean number of APs and simulation parameters underlying the bottom row of Figure 6B.)
• Figure_7_data.csv (Amplitudes, conductance scaling factors and firing rates underlying Figure 7.)
• Figure_8_data.csv (Mean number of APs and simulation parameters underlying Figure 8.)
• Figure_9_left_panel_data.csv (Mean firing rate and GKdr and GKM scaling factors underlying left panel of Figure 9.)
• Figure_9_right_panel_data.csv (Mean firing rate and GKdr and GKM scaling factors underlying right panel of Figure 9.)
• Equations_and_conductance_densities.docx (Equations and conductance densities for the baseline model.)

Data are available under the terms of the Creative Commons Attribution 4.0 International license.
