# Supplementary material for: A population of in silico models identifies the interplay between Nav 1.8 conductance and potassium currents as key in regulating human dorsal root ganglion neuron excitability
Source: F1000Res. 2022 Jan 27;11:104. [Version 1] doi: 10.12688/f1000research.74551.1 (PMC11406138; doi:10.12688/f1000research.74551.1)
Supplement: Peer review history

# Reviewer report: Reviewer response for version 1

**DOI:** 10.5256/f1000research.78322.r176219

**Published:** 2024-09-16

**Authors:** Joseph J Pancrazio

**Affiliations:** 1 The University of Texas at Dallas, Richardson, Texas, USA

The authors have created a novel approach to the utilization of Hodgkin-Huxley based models for excitable cells to interpret how pharmacological manipulation of underlying channels affects excitability. They do so by creating a diverse  "family" of permutations from a central model and parse the population by accepting those models that yield overall action potential behavior that offers parameters within the observation range of prior experiments. The authors focus on modeling the pharmacological inhibition of a particular channel type, Nav 1.8, which is implicated in the manifestation of chronic pain, and therefore, serves as a potential target for drug discovery.

 The approach is innovative yielding insight into the entanglement of voltage dependent ion channel activity and the action potential behavior that emerges and is also a function of that very ion channel behavior. The paper is well-written, the results are informative, yielding insight into degrees of channel inhibition that may -- just may be -- the right target level for effectiveness. I have relatively few comments for this innovative and impressive work:

 1. Fig. 4A showing a uniform grid with dots appears to be entirely uninformative. It appears to be simply indicating that there are 328 qualified models. I suggest removing Fig. 4A.

 2. "Implications for the 3Rs ''' section in the discussion. While well intentioned, the literature analysis which attempts to arrive at some number of animals lost in unnecessary experimentation is a weak argument. It seems to suggest that animal experimentation could have been eliminated if this modeling approach was adopted without reservation or further validation. While I am an optimist for the utility of modeling, the notion that the job is entirely done is premature. I suggest this section be eliminated and simply make the point that modeling of this nature has the potential to substantially reduce the animal subject burden during preclinical testing (as do hIPSCs for that matter).

Is the rationale for developing the new method (or application) clearly explained?

Yes

Are the 3Rs implications of the work described accurately?

Yes

Is the description of the method technically sound?

Yes

Are the conclusions about the method and its performance adequately supported by the findings presented in the article?

Yes

If any results are presented, are all the source data underlying the results available to ensure full reproducibility?

Yes

Are a suitable application and appropriate end-users identified?

Yes

Are sufficient details provided to allow replication of the method development and its use by others?

Yes

Reviewer Expertise:

microelectrode arrays, electrophysiology, nociceptors

I confirm that I have read this submission and believe that I have an appropriate level of expertise to confirm that it is of an acceptable scientific standard.

# Reviewer report: Reviewer response for version 1

**DOI:** 10.5256/f1000research.78322.r230124

**Published:** 2024-03-06

**Authors:** Markos Xenakis

**Affiliations:** 1 Institute of Neurophysiology, RWTH Uniklinik, RWTH Aachen University, Aachen, NRW, Germany

In the present work, the authors, Oliver J. Britton, and Blanca Rodriguez, make a decisive step toward whole-hDRG-neuron modeling and simulation. They are motivated by the need for more realistic insilico models for studying the transmission of nociceptive stimuli. They systematically investigate how inter-neuronal ion channel density heterogeneity contributes to the shape of hDRG action potential. This is done in a computationally efficient manner without sacrificing biophysics realism. Namely, the authors introduce a pool of hDRG models where model-to-model variability of ion channel conductances but not ion channel kinetics is implemented. Their modeling procedures are documented pedagogically. They emphasize the role of the NaV1.8 channel in shaping action potential properties. Specifically, they report on a balancing mechanism regulating neuron firing rate concerning an inflection point of the NaV1.8 conductance. Driving NaV1.8 conductance above its inflection point value decreases the neuron firing rate. The location of the inflection point is reported to depend on the delayed rectifier potassium conductance. The authors conclude that synergies among the NaV1.8 and other channels, especially potassium, play an essential role in modulating the NaV1.8 channel in nociceptive neurons.

 The study paves the way for the replacement, refinement, and reduction of animals in research. The presented model shows good potential in reproducing hDRG electrophysiological signatures. It is constructed comprehensively and transparently based on previous works and experimental data. What I missed, however, is a knowledge gain in neuropathic pain disease. Remarks and comments for improvement are attached below.

**Major comments**:

Methodological aspects:

 The authors do a great job documenting their procedures and sharing step-by-step guidelines on installing and running the software. However, all this occupies too much space in the manuscript, which could be used for other, more relevant purposes, such as explaining more in-depth how presented findings can help better understand neurophysiological aspects of nociception. I hence suggest that text and tables from p. 7 “Installing drgpom and compiling models .. ” to p. 13 “.. . This example can be used as a template to design your own simulation studies.” to be placed elsewhere. For example, the authors could consider the option of moving this material to the corresponding Zenodo and/or GitHub repository.

Results:

 On a few occasions, it is unclear whether findings reflect specific parameter choices or actual neurophysiological traits. A better explanation of the relationship between the former (parameter space) and the latter (neurophysiology) could help. Specifically:

 “For Gnav 1.8, this is because models with low GNav 1.8 have peak voltages that are below the experimental range, and models with high GNav 1.8 have threshold voltages that are below the experimental range. For GKA, no model with high GKA had a combination of threshold voltage, peak voltage, and after-hyperpolarisation time constant that was simultaneously within the experimental ranges of these three AP biomarkers.”

 - What is the neurophysiological rationale in support of these simulation results?

 “This correlation is caused by calibrating on a combination of three biomarkers: threshold voltage, peak voltage and AP full width parameters, as removing all of these AP biomarkers from the calibration process also removes the correlation between GNav 1.8 and GKdr and removing any one of them reduces the correlation coefficient. This indicates that in our model the balance of these two currents is important throughout the action potential, from upstroke (threshold, peak voltage) to repolarisation (AP full width).”

 - What is the principle underlying reported correlation? Is it a computational artifact, or does it reflect a biophysically interesting phenomenon?

 “The relative proportions of these currents are however highly variable from model to model. Similarly, the main currents during the upstroke are INav 1.7 (red) and INav 1.8 (blue), but some models have a large contribution by Nav 1.7 and in some models the contribution is very small, while the contribution of INav 1.8 is uniformly large.”

 - Could the authors clarify how NaV1.7 and NaV1.8 contribute differently to the current generation?

Conclusions and Discussion:

 Based on the abstract and keywords, the reader expects to learn something about neuropathic pain disease. After reading the manuscript, however, this expectation still needs to be met. The role of the NaV1.7, which has emerged as a key regulator of neuropathic pain disease, could also be addressed, especially concerning the pivotal role NaV1.8 is suggested to have here.

 Could the authors elaborate on how presented findings and modeling procedures enhance our understanding of neuropathic pain?

**Minor comments:**

 On the first page, in the Methods description section, a full stop is missing: “.. in ionic conductances and action potential morphology” should read “.. in ionic conductances and action potential morphology.”

- p. 6: Please provide SI units of the time constant \tau
- p. 9: “Temperature in Celcius” should read “Temperature in Celcius”
- p. 12: “Davidson et al., 2014 PAIN are supported”. Is “PAIN” a typo here?
- p. 13: “.. consisted of 328 models .. “. Where does the number 328 comes from?
- p. 14: “For GNav 1.8, this is because models with low GNav 1.8 have peak voltages that are below the experimental range, and models with high GNav 1.8 have threshold voltages that are below the experimental range." This is unclear and needs clarification (see also major comment above).
- p. 16: “As we show later, the balance of these currents is not as critical as the balance of GNav 1.8 and GKdr at rheobase, where only a single AP is fired.” Please clarify what “critical” means in this context.
- Figure 4A: Is this illustration really necessary?
- p. 22: “.. almost complete .. “ should read “.. almost completely ..”
- p. 24: “Similar approaches to this have been used .. ” should read “Similar approaches have been used ..”
- p.24: Tigerholm et al. (2015) and Choi and Waxman (2011) hyperlinks do not work.

Is the rationale for developing the new method (or application) clearly explained?

Yes

Are the 3Rs implications of the work described accurately?

Yes

Is the description of the method technically sound?

Yes

Are the conclusions about the method and its performance adequately supported by the findings presented in the article?

Partly

If any results are presented, are all the source data underlying the results available to ensure full reproducibility?

Yes

Are a suitable application and appropriate end-users identified?

Yes

Are sufficient details provided to allow replication of the method development and its use by others?

Yes

Reviewer Expertise:

Computational Neurophysiology

I confirm that I have read this submission and believe that I have an appropriate level of expertise to confirm that it is of an acceptable scientific standard, however I have significant reservations, as outlined above.

# Reviewer report: Reviewer response for version 1

**DOI:** 10.5256/f1000research.78322.r208694

**Published:** 2023-12-14

**Authors:** Stewart Heitmann

**Affiliations:** 1 Victor Chang Cardiac Research Institute, Sydney, New South Wales, Australia

This study uses computational modelling to investigate how the human dorsal root ganglion (DRG) neuron responds when the Nav 1.8 sodium channel is selectively modulated. The study is motivated by the role of the DRG  neuron in nociception (pain) and the role of the Nav 1.8 channel in modulating the excitability of that neuron. A population of models is used to investigate phenotypically diverse DRG neurons drawn from the human population. They find that the excitability of the neuron depends not only on Nav 1.8 but also on potassium ion channels which appear to be naturally co-regulated with Nav 1.8.

 The proposed model offers practical benefits in reducing the use of animals in pain research, which I applaud. The population of models approach is useful for addressing the issue of natural variation too.

**Methods**

 A substantial part of the manuscript is dedicated to instructions for installing and running the software. Documenting the software is important, but those issues are separate from the scientific question that is being investigated (about ion channels). So I urge the authors to move all of the software-related text (pages 7-13) into a separate software manual or tutorial paper. Software documentation is best shipped with the source code.

**Discussion**

 Upon reading the discussion, I felt no wiser about how co-regulated INav 1.8 and potassium currents might inform better treatments for patients with pathological pain. The discussion of the M-type potassium current seemed to hint that it might it be possible to treat a defective Nav 1.8 ion channel by targeting the potassium channels. I would have like to see that sort of thing explored in greater depth.

**Minor Comments**

 pg 3. Suggested rewording:

*“For example, a gain-of-function mutation in Nav 1.7 caused hyper-excitability in DRG neurons that expressed Nav 1.8, yet the same mutation caused hypo-excitability in DRG neurons that lacked Nav 1.8 {Rush et al., 2006]”*

 pg 4. “… with eight action potential parameters.” *Biomarkers* is a better word than *parameters*.

 pg 4. “In this study, we use a non-standard modelling approach … “.  Is it really non-standard?  Perhaps say instead: “In this study, we use a modelling approach based on experimentally calibrated …”  

 pg 4. “We developed a baseline model …”

 I suggest moving Figure 1B here to support the text.

 p5. “The equations for the baseline model can be found in the supplementary material”.

 Personally, I like to see the equations included in the main text, if only in abbreviated form, so that the reader can appreciate what is being modeled. However, that is not always practical.

 p6. “Each model was simulated …”

 It would be nice to plot the stimulus protocols to spare the reader from looking it up. It is also worth describing how the randomized models were equilibrated prior to being analyzed. Some AP models can take hundreds of beats to equilibrate to a new parameter regime.

 p14. “This correlation is caused by calibrating on …”

 I’m not sure this is the best wording. Instead of saying the correlation is *caused* by the calibration (which is to say that it is caused by your method), I suggest saying that the correlation reveals the normal relationship between ion channels (as caused by nature).

 Figure 3A. The **a-h** labels on the abscissa are slightly confusing. Better to label them as GNav17, GNav18, etc.

 Figure 4A is unneccesary. You could omit it altogether. The idea is clear enough from panels B and C. That said, the effect of Nav 1.8 scaling could be made even clearer by plotting it for only one stimulus amplitude, say 4000pA.

 p18. “We hypothesised that one reason for the decrease in firing rate …”.

 Illustrating the idea with some example AP trains would be helpful.

 p25. “To estimate the number of animals currently …”

 There is no need to explain the details of the pubmed search, just summarize it. For example: *“To estimate the number of animals that could be potentially be replaced with our proposed DRG model, we searched pubmed for all papers in the past five years that described pain research using DRG neurons in rodents. We estimated that 21,000 animals were used annually for this purpose. These estimates do not include animal use in industry”.*

Is the rationale for developing the new method (or application) clearly explained?

Yes

Is the description of the method technically sound?

Partly

Are the conclusions about the method and its performance adequately supported by the findings presented in the article?

Partly

If any results are presented, are all the source data underlying the results available to ensure full reproducibility?

Yes

Are sufficient details provided to allow replication of the method development and its use by others?

Yes

Reviewer Expertise:

Computational cardiology

I confirm that I have read this submission and believe that I have an appropriate level of expertise to confirm that it is of an acceptable scientific standard, however I have significant reservations, as outlined above.

# Reviewer report: Reviewer response for version 1

**DOI:** 10.5256/f1000research.78322.r208690

**Published:** 2023-12-13

**Authors:** Igor Vorobyov, Dr. Gonzalo Hernandez Hernandez

**Affiliations:** 1 Department of Physiology and Membrane, University of California Davis, Davis, California, USA; 2 University of California Davis, Davis, CA, USA

I have reviewed the manuscript in detail and recommend it for **approval with specific reservations**. While the paper demonstrates considerable academic merit, there are minor modifications that, if addressed, could enhance the overall strength and impact of the article.

**Summary:**

 This manuscript by Britton O.J and Rodriguez B.  represents a comprehensive method article introducing an open-source software designed for simulating human dorsal root ganglion (hDRG) neuron electrophysiology. Initially, a baseline model with eight transmembrane currents representing sodium and potassium channels, was established, followed by the generation of a pool of candidate models using a population approach. This aimed to align them with specific experimental AP biomarker ranges. In addition, the research focused on the Na _V_1.8 channel conductance to elucidate its influence on neuronal excitability in relation to other ion channels in nociceptive neurons. Notably, modifications in the Na _V_1.8 conductance revealed an interesting behavior: a moderate increase heightened the firing rate, but an excessive increase reduced it. The study also underscored the importance of delayed rectifiers and M-type potassium conductances in determining neuronal excitability. In particular, perturbations in the delayed rectifier potassium conductance affected the Na _V_1.8's impact on the firing rate. The authors performed a detailed simulation analysis on the variation of those as well as other conductances on the action potential firing rates revealing interesting trends, which can be tested experimentally and, in the future, also used for the development of new specific ion channel modulators.

**Strengths:**

 The manuscript is well written. The hypothesis is well-defined and the rationale for the study is clear, giving readers a good idea of what to expect in the subsequent sections. In addition, the methodology employed for capturing inter-neuronal variability is noteworthy.

 The model accounts for variations in ionic conductances and action potential morphology, making it more comprehensive and likely more representative of biological reality.

 A major strength of the paper is the sharing of their software openly. This move makes the research more trustworthy and lets others build on it. Open tools can also help new researchers learn. Instructions for the use of software are very clear.

**Areas of potential improvement:**

 By focusing solely on the soma, the model may not capture the complete electrophysiological behavior of the entire neuron, especially any interactions between the soma and an axon. This is perhaps beyond the scope of the study and can be acknowledged as a study limitation.

 The simplifying assumption that ion channel structures do not vary between different DRG neurons might not hold true for all neurons and conditions. There is ion channel structural variability, which may influence their open probabilities, gating time constants and other functional parameters. This assumption could impact the overall accuracy and applicability of the model and can be also mentioned as a limitation.

 I would also consider moving sections of the Methods, which discuss software installation, running etc. towards the end of the paper as an Appendix, in order not to break the flow of the paper. 

 Upon a detailed evaluation of your manuscript, I appreciate the depth of the research presented. I believe that with certain refinements in the figures, Results, and Discussion sections, the manuscript will greatly benefit in its readability and overall impact.

 Here are my specific recommendations:

  

1. Figure 1. The current name on the right got cut. Analysis: It remains ambiguous if these models were run until they achieved a particular steady-state and then fitted it to the mean values of each parameter based on the data recorded from *ex vivo* hDRG neurons.
2. For Figure 2, utilizing smaller data points and a more distinguishable color palette could enhance the clarity of the distribution of conductances across the population.
3. Figure 3, The "currentscape" visualisation technique implemented in Figure 3 offers a unique insight into the conductances and relative current magnitudes across six models. To further its effectiveness: Opt for color schemes that offer greater contrast to discern the various currents more distinctly. Incorporate a dynamic repetition of action potentials to enrich presentation.
4. Figure 4. I am not sure if panel A is needed, as the same information is already present in panels B and C, as the same range and individual data points as squares are shown there. Although, I do see that Fig. 4A is referenced multiple times for other data as well. 
5. Figure 5. top panel. No action potential (AP) firing is visible. Maybe, an inset with zoomed in data on this panel would be helpful.
6. Figure 6. What is the difference between dark- and light-red plots in panel A?
7. Figures 7 & 8: Both figures summarise the principal trends recognized in the study. To augment the easiness of their interpretation: Enhance the legibility of the *x* and *y* axes, the axes are hard to read as the figure contains multiple panels.
8. Figure 9 Colors for GKM/GKdr 0 and 2 as well as for 0.5 and 1.5 are hard to distinguish.
9. While the procedure involving the stimulation of different levels of block and enhancement of Na _V_1.8 conductance is mentioned, a clearer description of the methodology could be helpful. This includes specifics about how 'different levels' were defined and chosen. Currently it is not clear whether they (as well as other conductances) were selected solely based on physiological interneuron variability, action of a pharmaceutical or another modulator or both.
10. The Na _V_1.8'’s influence on hDRG neuron excitability analysis could benefit from a section discussing potential future investigations or implications of these findings on possible therapeutic interventions, given the evident role of Na _V_1.8 in neuronal firing.
11. The comparison and usage of previous modeling studies of DRG neurons, particularly from Tigerholm et al. and Choi and Waxman, adds depth to the study. It would be advantageous to highlight any adaptations or modifications made to these referenced studies' methodologies and the reasoning behind these changes.
12. Throughout the paper: It would be best not to use space in the "GNav 1.8” and similar variables. Ideally one would also use subscript for channel name, i.e., "G_{Nav1.8}". Ideally, voltage-gated ion channel names should include subscript capital “V” and not “v” as well.
13.  Please briefly explain the word "rheobase", which is specific to the ion channel electrophysiology field and may not be known to a general reader.
14.  Please explain what IKdr current is.
15. There is no link for "Choi and Waxman, 2011" paper.
16. "Each cells containing code" -> "Each cell containing code"
17. "kleak ion channel model " -> "leak K^+ channel (Kleak) model"?
18. "simulating changes in multiple conductances at multiple different levels" - Do you mean levels associated with certain changes of conductances from the base model or something else?
19. "For GNav 1.8, this is because models with low GNav 1.8 have peak voltages that are below the experimental range, and models with high GNav 1.8 have threshold voltages that are below the experimental range."  - please check if this is correct as stated, as it seems that both low and high "GNav 1.8 " have the same effect.
20. "combination of three biomarkers: threshold voltage, peak voltage and AP full width parameters". Are these parameters? I thought they were properties or simulation outputs.
21. ""That GKdr is actually larger in the rapidly firing group suggests it is not just a lack of total potassium current conductance, and a corresponding lack of outward current to oppose repetitive firing, that causes these models to fire so rapidly." This sentence is confusing, please clarify if possible. Maybe, the comma after "conductance" causes this confusion. 
22. "Mean animals used for DRG studies in the 16/40 papers where number of animals used was reported or could be inferred from figures was 103". Please reword this sentence as it is confusing from the start. What does  "mean animals" mean?

**Conclusions: **The authors have done an excellent job in pointing out areas for potential improvements in their model. Adding more details about calcium-related currents and pumps and transporters will make the model even better. The authors also did not mention chloride channels, which are known to be present in DRG neurons as well (see e.g., Wilke et al Front Neurosci. 2020; 14: 287). Also, showing the full structure of hDRG neurons, such as the connections between axons and the main cell body (soma), will make the model more realistic. I look forward to seeing how the authors will refine their work in future updates and how they will extend their population models to capture hDRG neuron variability in human datasets available in the future.

Is the rationale for developing the new method (or application) clearly explained?

Yes

Are the 3Rs implications of the work described accurately?

Yes

Is the description of the method technically sound?

Yes

Are the conclusions about the method and its performance adequately supported by the findings presented in the article?

Yes

If any results are presented, are all the source data underlying the results available to ensure full reproducibility?

Yes

Are a suitable application and appropriate end-users identified?

Yes

Are sufficient details provided to allow replication of the method development and its use by others?

Yes

Reviewer Expertise:

Ion channel and G protein-coupled receptor molecular modeling and simulations.

We confirm that we have read this submission and believe that we have an appropriate level of expertise to confirm that it is of an acceptable scientific standard, however we have significant reservations, as outlined above.
